# AlkB RNA demethylase homologues and *N* ^6^ ‐methyladenosine are involved in *Potyvirus* infection

**DOI:** 10.1111/mpp.13239

**Published:** 2022-06-14

**Authors:** Jianying Yue, Yao Wei, Zhenqi Sun, Yahan Chen, Xuefeng Wei, Haijuan Wang, Fabio Pasin, Mingmin Zhao

**Affiliations:** ^1^ College of Horticulture and Plant Protection Inner Mongolia Agricultural University Hohhot China; ^2^ College of Plant Protection Gansu Agricultural University Lanzhou China; ^3^ Development of Fine Chemicals Guizhou University Guizhou China; ^4^ Instituto de Biología Molecular y Celular de Plantas (IBMCP) Consejo Superior de Investigaciones Científicas—Universitat Politècnica de València (CSIC‐UPV) Valencia Spain; ^5^ School of Science University of Padua Padua Italy

**Keywords:** alkylation B (AlkB), RNA methylation, *N* ^6^‐methyladenosine (m^6^A), plant–virus interaction, *Potyvirus*, endive necrotic mosaic virus (ENMV)

## Abstract

Proteins of the alkylation B (AlkB) superfamily show RNA demethylase activity removing methyl adducts from *N* ^6^‐methyladenosine (m^6^A). m^6^A is a reversible epigenetic mark of RNA that regulates human virus replication but has unclear roles in plant virus infection. We focused on *Potyvirus*—the largest genus of plant RNA viruses—and report here the identification of AlkB domains within P1 of endive necrotic mosaic virus (ENMV) and an additional virus of a putative novel species within *Potyvirus*. We show that *Nicotiana benthamiana* m^6^A levels are reduced by infection of plum pox virus (PPV) and potato virus Y (PVY). The two potyviruses lack AlkB and the results suggest a general involvement of RNA methylation in potyvirus infection and evolution. Methylated RNA immunoprecipitation sequencing of virus‐infected samples showed that m^6^A peaks are enriched in plant transcript 3′ untranslated regions and in discrete internal and 3′ terminal regions of PPV and PVY genomes. Down‐regulation of *N. benthamiana* AlkB homologues of the plant‐specific ALKBH9 clade caused a significant decrease in PPV and PVY accumulation. In summary, our study provides evolutionary and experimental evidence that supports the m^6^A implication and the proviral roles of AlkB homologues in *Potyvirus* infection.

## Graphical abstract

AlkB RNA demethylases remove methyl adducts from *N* ^6^‐methyladenosine. We provide evolutionary and experimental evidence that supports the proviral roles of AlkB homologues in *Potyvirus* infection.

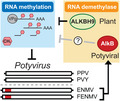

Host–virus interaction is complex and our comprehension of the underlying molecular mechanisms is far from complete. Recent sequencing efforts coupled with novel bioinformatic pipelines have uncovered an unprecedented number of immune systems involved in prokaryotic antiviral responses (Bernheim & Sorek, 2020; Gao et al., 2020). Equivalent or higher abundance of immune system components is predicted to occur in multicellular organisms, including plants (Alazem & Lin, 2015; Li & Wang, 2019; Ngou et al., 2022; Wang et al., 2022).

RNA methylation at internal positions is a reversible epigenetic mark that regulates almost all RNA biology aspects such as RNA processing, maturation and decay, nuclear export, and translation (Liang et al., 2020; Shi et al., 2019). *N* ^6^‐methyladenosine (m^6^A) is among the most abundant of the over 160 known RNA modification types. m^6^A is widely distributed in prokaryotic and eukaryotic transcriptomes and its abundance is dynamically regulated by cellular enzymes with RNA methyltransferase (writer) or demethylase (eraser) activity (Schauerte et al., 2021; Shi et al., 2019; Zhang, Qian, et al., 2021).

It has been known since the 1970s that m^6^A occurs in viral RNAs (Lavi & Shatkin, 1975; Yue et al., 2022; Zhang, Qian, et al., 2021), but advances in its detection and mapping are redefining our understanding of the roles of this modification in virus infection (Dang et al., 2019; Zhang, Qian, et al., 2021). Genetic manipulation of m^6^A writer or eraser levels has enabled the establishment of models in which m^6^A acts as a proviral modification that, for instance, promotes replication of influenza A virus (Courtney et al., 2017) and human immunodeficiency virus‐1 (Lichinchi, Gao, et al., 2016). Other data suggest that m^6^A may participate in antiviral immunity as it was shown to negatively regulate hepatitis C virus (Gokhale et al., 2016), Zika virus (Lichinchi, Zhao, et al., 2016), vesicular stomatitis virus (Zheng et al., 2017), and severe acute respiratory syndrome coronavirus 2 (Liu et al., 2021), among others.

In plants, m^6^A functionally modulates a variety of processes including development and responses to biotic and abiotic stress (Zhou et al., 2022). Very recent reports support its involvement in plant–virus interaction (Yue et al., 2022). Enzymes of the alkylation B (AlkB) superfamily reverse m^6^A methylation (Fedeles et al., 2015), and AlkB domains are present within viral replication‐associated proteins of plant RNA viruses in the families *Closteroviridae*, *Alphaflexiviridae*, *Betaflexiviridae*, and *Secoviridae* (Bratlie & Drabløs, 2005; Halgren et al., 2007; Moore & Meng, 2019). m^6^A has been identified in the RNA genome of alfalfa mosaic virus (genus *Alfamovirus*). It has been suggested to be part of a novel antiviral system because genetic depletion of a plant m^6^A demethylase negatively affects alfalfa mosaic virus accumulation and movement (Martínez‐Pérez et al., 2017, 2021). The overall plant m^6^A level has been reported to be altered by RNA viruses of the genera *Tobamovirus*, *Bymovirus, Tenuivirus*, and *Fijivirus* (He et al., 2021; Li et al., 2018; Zhang, Zhuang, et al., 2021; Zhang, Wang, et al., 2021). Despite these recent studies, the biological significance of internal RNA methylation and m^6^A in plant virus infection and antiviral immunity is poorly understood.

Here, to advance our knowledge of RNA methylation involvement in plant–virus interaction, we focused on members of the *Potyvirus* genus, the largest group of plant RNA viruses and part of the *Potyviridae* family. Besides *Potyvirus*, *Potyviridae* currently comprises the additional 11 genera *Arepavirus*, *Bevemovirus*, *Brambyvirus*, *Bymovirus*, *Celavirus*, *Ipomovirus*, *Macluravirus*, *Poacevirus*, *Roymovirus*, *Rymovirus*, and *Tritimovirus* (Gibbs et al., 2020; Pasin et al., 2022).

Potyvirid genomes are translated into large polyproteins with a conserved core and divergent leaders (Pasin et al., 2022). A viral AlkB domain with RNA demethylase activity was reported in the polyprotein leader of blackberry virus Y (BlVY), an atypical potyvirid of *Brambyvirus* (van den Born et al., 2008; Susaimuthu et al., 2008). We asked if absence of additional reports of potyvirid AlkB homologues is due to incomplete annotations of available genomic resources. To comprehensively identify functional domains within potyvirid proteins (Table S1; Supporting Methods of File S1), complete or near‐complete genomic sequences of recognized species were scanned using position‐specific scoring matrix and hidden Markov model protein profiles from the Prosite, SUPERFAMILY, and Gene3D libraries (Lewis et al., 2018; Sigrist et al., 2013; Wilson et al., 2009). Our family‐wide approach detected the previously described BlVY domain. As very recently reported (Pasin et al., 2022), it further uncovered an AlkB homologue in the GenBank accession KU941946, annotated as endive necrotic mosaic virus (ENMV) within the *Potyvirus* genus (Desbiez et al., 2017). A limited number of recognized members of *Potyviridae* (2/185), and 0.7% of *Potyvirus* members (1/142) thus encode AlkB (Figure 1a).

**FIGURE 1:**
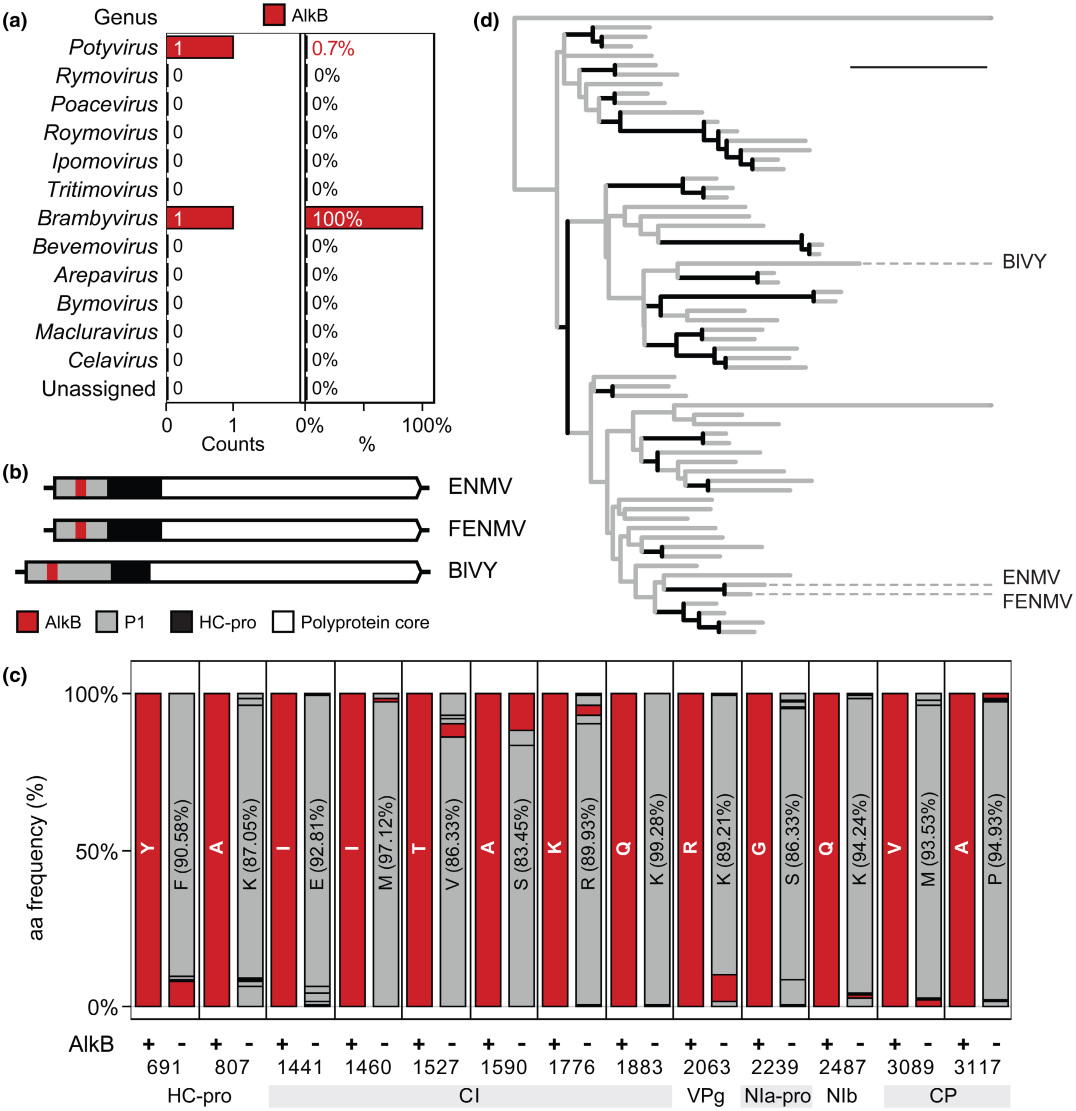
Alkylation B (AlkB) is encoded by two members of the *Potyvirus* genus. (a) AlkB abundance in recognized species of the *Potyviridae* family was obtained by protein profiling (Supporting Methods of File S1; Table S1). Absolute numbers (counts) and counts per species (%) are shown for each genus. Unassigned includes common reed chlorotic stripe virus, Spartina mottle virus, and longan witches' broom‐associated virus. (b) Diagrams of potyvirid genomes encoding AlkB. RNA and polyproteins are represented as lines and arrowed boxes, respectively, and relevant domains are labelled. ENMV, endive necrotic mosaic virus (*Potyvirus*; OM867853); FENMV, French endive necrotic mosaic virus (*Potyvirus*; KU941946); BlVY, blackberry virus Y (*Brambyvirus*; AY994084). (c) Polyprotein variants significantly overrepresented in (F)ENMV. At each position, the consensus residue in AlkB‐encoding isolates is shown in red, and the amino acid frequency of genus members lacking AlkB is shown. Polyprotein site numbers refer to ENMV (UOF93311); see Table S3 for statistics. (d) Evolutionary divergence of potyvirid AlkB. Domains of plant viruses were aligned, and phylogeny was inferred using *Escherichia coli* AlkB as an outlier. (F)ENMV and BlVY are labelled; tree branches with bootstrap support ≥80 are shown in black. Scale bar = 1. Protein accession numbers and residue positions are given in Table S4.

ENMV and its coat protein (CP) sequence AJ223827 were first reported in the 1990s by H.‐J. Vetten (Germany). AJ223827 shares low nucleotide identity (79.19%) to the genomic accession KU941946 later obtained in France (Desbiez et al., 2017). To better assess ENMV diversity, we retrieved near‐complete genomic sequences (OM867853, OM867861) of progeny of two isolates originally obtained by H.‐J. Vetten. As expected, both sequences share high nucleotide identity to AJ223827 (97.85%–97.92%) and can be considered as ENMV reference genomes. NCBI database searches using these sequences additionally identified LC631971 and LC631972, genomes obtained in Australia and annotated as endive yellows virus. Pairwise comparisons of polyproteins and coding sequences show that isolates from Germany and Australia share (i) high identity with each other (nucleotide sequences: 83.2%–99.9%; amino acid sequences: 91.9%–100.0%), indicating they belong to a single species, and (ii) identity values of only 70.9%–71.6% (nucleotide sequences) and 77.6%–77.8% (amino acid sequences) with the French isolate (Tables 1 and 2), which are below the family's species demarcation thresholds (nucleotide sequences: 76%; amino acid sequences: 82%) (Inoue‐Nagata et al., 2022). We thus refer here to KU941946 as French endive necrotic mosaic virus (FENMV), member of a putative new species within the *Potyvirus* genus.

**TABLE 1:** Pairwise identity values of polyprotein coding sequences of ENMV isolates and reference viruses

Virus			Nucleotide identity (%)^a^						
Name	Acc. no.	Nucleotides	AY994084	KF906523	KU941946	LC631971	LC631972	OM867853	OM867861
BlVY	AY994084	177–10,652	**100.0**						
VDMV	KF906523	102–9353	52.6	**100.0**					
FENMV	KU941946	124–9834	52.0	61.9	**100.0**				
ENMV	LC631971	107–9829	53.5	61.7	71.6	**100.0**			
ENMV	LC631972	107–9829	52.8	61.5	71.6	**98.7**	**100.0**		
ENMV	OM867853	124–9846	52.2	61.4	70.9	**83.2**	**83.2**	**100.0**	
ENMV	OM867861	124–9846	52.2	61.4	70.9	**83.2**	**83.2**	**99.9**	**100.0**

Abbreviations: BlVY, blackberry virus Y; ENMV, endive necrotic mosaic virus; FENMV, French endive necrotic mosaic virus; VDMV, vanilla distortion mosaic virus.

^a^ Values above species demarcation thresholds are shown in bold.

**TABLE 2:** Pairwise identity values of polyprotein sequences of ENMV isolates and reference viruses

Virus			Amino acid identity (%)^a^						
Name	Acc. no.	Amino acids	AAX87001	AHU88030	ARF07717	BCW03298	BCW03299	UOF93311	UOF93331
BlVY	AAX87001	1–3491	**100.0**						
VDMV	AHU88030	1–3083	31.8	**100.0**					
FENMV	ARF07717	1–3236	31.1	59.8	**100.0**				
ENMV	BCW03298	1–3240	30.9	59.7	77.6	**100.0**			
ENMV	BCW03299	1–3240	30.9	60.1	77.8	**98.7**	**100.0**		
ENMV	UOF93311	1–3240	31.9	60.5	77.8	**91.9**	**92.3**	**100.0**	
ENMV	UOF93331	1–3240	31.8	60.5	77.8	**92.0**	**92.3**	**100.0**	**100.0**

Abbreviations: BlVY, blackberry virus Y; ENMV, endive necrotic mosaic virus; FENMV, French endive necrotic mosaic virus; VDMV, vanilla distortion mosaic virus.

^a^ Values above species demarcation thresholds are in bold.

AlkB was identified with high confidence in all (F)ENMV isolates by the SUPERFAMILY and Gene3D hidden Markov model profiles (significance score ≤ 8.5 × 10^−20^; Table S2); residues critical for catalysis are conserved in the identified domains (Figure S1). No AlkB was detected in vanilla distortion mosaic virus, a potyvirus close to FENMV and ENMV (Table S2; Tables 1 and 2). Potyvirid AlkB domains are all embedded in P1 (Figure 1b), a leader proteinase with conserved structural disorder at its amino terminus (Pasin et al., 2014). AlkB presence in BlVY was associated with a truncated HC‐Pro of 325 amino acids (Susaimuthu et al., 2008). Polyprotein comparisons between (F)ENMV and reference *Potyvirus* members could not detect major structural variation beyond P1; specifically, the (F)ENMV HC‐Pro length of 458 amino acids matches the mean value of 458.1 ± 8.9 of homologues from potyviruses lacking AlkB (*n* = 141). Primary sequence compositional analysis of the genus nonetheless identified HC‐Pro, CI, VPg, NIa‐Pro, NIb, and CP residues that were specific or significantly over‐represented in AlkB‐encoding isolates (Figure 1c; Table S3). Most variants were located within CI, an RNA helicase involved in viral replication and cell‐to‐cell movement (Revers & García, 2015). Biological significance of these findings remains to be determined.

Phylogeny of potyvirid AlkB domains was inferred alongside plant virus homologues to investigate their evolution. The obtained tree showed that domains of (F)ENMV share a monophyletic origin, whereas BlVY AlkB belongs to a significantly divergent branch (Figure 1d; Table S4). The results show that (i) two potyviruses and a brambyvirus encode AlkB, (ii) at least two independent gene acquisition events have participated in potyvirid AlkB evolution, and (iii) RNA methylation is a likely evolutionary driver of *Potyvirus*.

m^6^A is a mayor epigenetic RNA mark in plants and a substrate of AlkB RNA demethylases (Fedeles et al., 2015; Liang et al., 2020). We focused on m^6^A to evaluate the general involvement of RNA methylation in potyvirus infection. Plum pox virus (PPV) and potato virus Y (PVY), two model potyviruses with no AlkB, were inoculated to *Nicotiana benthamiana* plants and upper uninoculated leaves were collected at 14 days postinoculation (dpi). Total RNA samples from virus‐infected and healthy plants were obtained and m^6^A levels were determined by an ELISA‐like assay based on an m^6^A‐specific antibody. Quantification results showed that m^6^A levels were significantly reduced in PPV‐ and PVY‐infected samples compared to healthy controls (Figure 2a). In agreement with our findings, a decrease in m^6^A level was previously observed in tobacco plants infected with an RNA virus of *Tobamovirus* (Li et al., 2018).

**FIGURE 2:**
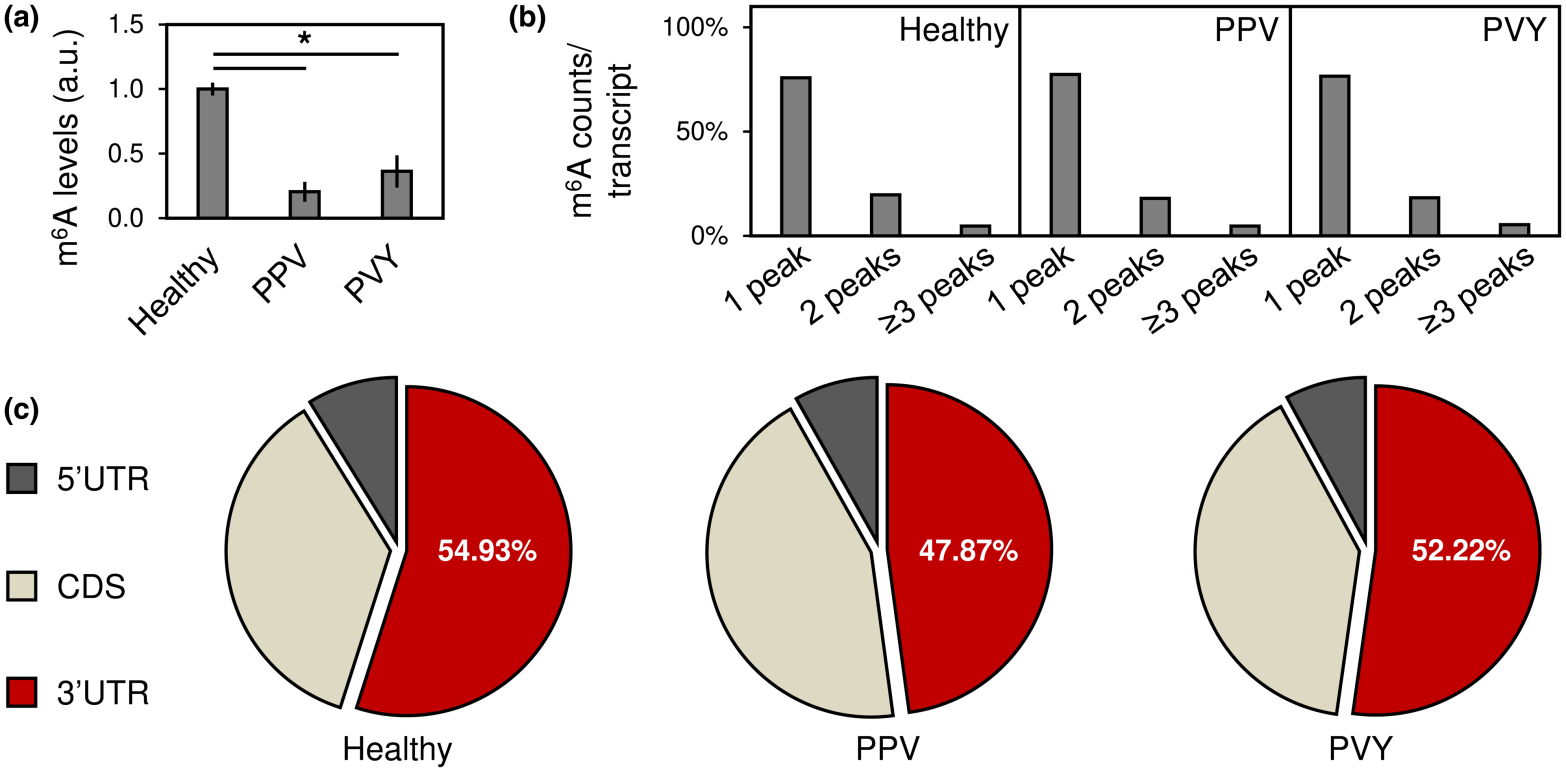
*N* ^6^‐methyladenosine (m^6^A) abundance and distribution in *Nicotiana benthamiana* transcriptomes upon potyvirus infection. Healthy *N. benthamiana* plants or those infected with plum pox virus (PPV; *Potyvirus*) and potato virus Y (PVY; *Potyvirus*) were analysed. (a) m^6^A amount in total RNA samples was quantified by ELISA; values are plotted as mean ± standard deviation; *p < 0.05 by Student's *t* test. (b) m^6^A‐containing RNAs were enriched by immunoprecipitation and sequenced (MeRIP‐seq). Percentages of enriched plant transcripts with single or multiple m^6^A peaks are shown. (c) Plots show distribution of MeRIP‐seq identified peaks across 5′ untranslated regions (UTRs), coding sequences (CDSs), and 3′ UTRs of plant transcripts.

We performed a methylated RNA immunoprecipitation sequencing (MeRIP‐seq) experiment to better characterize host transcriptome‐wide m^6^A alteration upon potyvirus infection. Poly(A) molecules were enriched from total RNA samples obtained from three biological replicates and then immunoprecipitated with an m^6^A‐specific antibody. The recovered immunoprecipitated RNA fragments were sequenced alongside input control samples to yield ≥3.2 × 10^7^ clean reads/sample (Table S5); these were mapped to a reference *N. benthamiana* genome. Bioinformatics revealed that approximately 76% of m^6^A‐containing transcripts had a single methylation peak, whereas occurrence of multiple m^6^A peaks within the same transcript was less frequent in both healthy and infected conditions (Figure 2b). In plants, transcript m^6^A marks are preferentially deposited around stop codons and within 3′ untranslated regions (3′ UTRs). This m^6^A distribution appears to be evolutionarily conserved in crops and divergent plant species (Miao et al., 2022; Zhou et al., 2022). Consistent with these reports, approximately 50% of m^6^A peaks identified in both healthy and virus‐infected samples were located in host transcript 3′ UTRs (Figure 2c).

We next asked if, besides plant transcripts, potyvirus RNA molecules (Figure 3a) could present m^6^A modifications. In silico analysis predicted multiple putative m^6^A sites within PPV and PVY genomes, albeit with varying confidence degrees (Figure S2). To experimentally validate the finding, MeRIP‐seq reads were mapped to PPV and PVY genomes and processed to identify m^6^A peaks. Our bioinformatic pipeline identified two and four m^6^A peaks significantly enriched in PPV and PVY genomes, respectively (Figure 3b; Table S6). Inspection of peak genomic positions indicated that m^6^A was preferentially deposited within virus genome 3′ termini, consistent with host transcriptome results.

**FIGURE 3:**
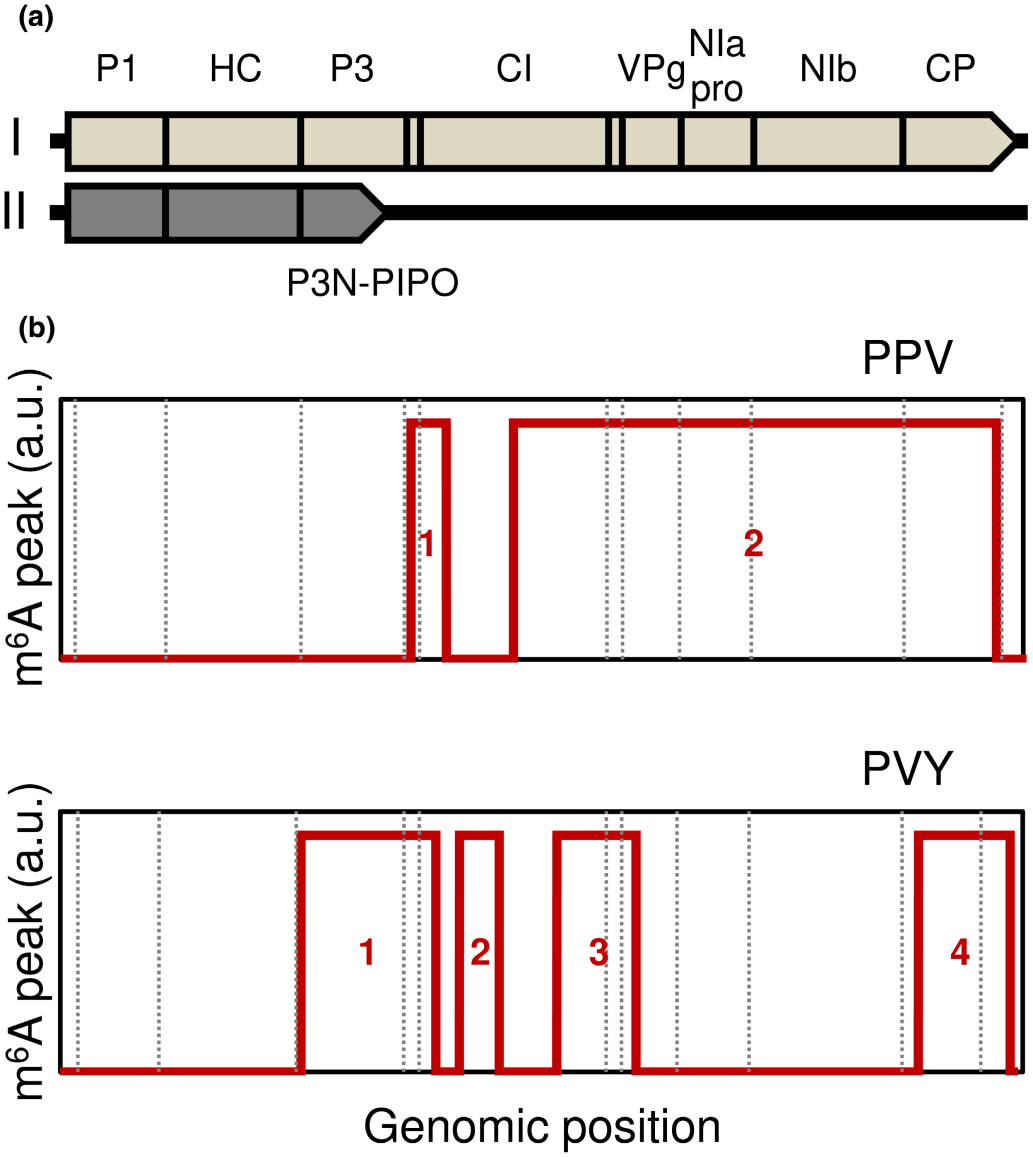
m^6^A peaks in internal and 3′ terminal regions of PPV and PVY genomes. (a) Diagram of potyvirus RNA molecules. RNA and encoded polyproteins are represented as lines and arrowed boxes, respectively, and relevant domains are labelled; full‐length polyprotein is shown (I), as well as the truncated polyprotein generated by P3 frameshifting (II). (b) Distribution of the identified methylated RNA immunoprecipitation sequencing peaks across PPV (top) and PVY (bottom) genomes is shown; dotted lines indicate the polyprotein cistrons shown in (a).

Slippage of the potyvirus replicase causes frameshifting in the P3 cistron and synthesis of P3N‐PIPO‐encoding transcripts characterized by premature stop codons and large 3′ UTRs (Figure 3a) (Yang et al., 2021). The identified PPV and PVY peaks 1 are located within or immediately downstream from the P3N‐PIPO cistron (Figure 3b). m^6^A involvement in P3N‐PIPO expression dynamics including transcription slippage and translation will require further investigation. It is also unclear if m^6^A could be enriched within or downstream from the coding region of PISPO, a potyvirus cistron generated by P1 frameshifting and premature polyprotein termination (Mingot et al., 2016; Rodamilans et al., 2021; Untiveros et al., 2016).

m^6^A levels are dynamically regulated by an activity balance of RNA methyltransferases and demethylases (Shi et al., 2019). m^6^A deposition is performed by a multicomponent complex with a conserved core alongside additional adaptor and modulator proteins not fully characterized in plants (Liang et al., 2020). m^6^A removal is in turn mediated by single‐component enzymes of the AlkB superfamily (Fedeles et al., 2015). RNA demethylase activity was confirmed in vitro or in vivo for AlkB homologues from plants and viruses (van den Born et al., 2008; Duan et al., 2017; Martínez‐Pérez et al., 2017; Zhou et al., 2019). *Arabidopsis thaliana* has 14 AlkB homologues, including members of the specific clades ALKBH9 and ALKBH10 that are not found in metazoans (Kawai et al., 2014; Mielecki et al., 2012). A putative AlkB RNA demethylase gene is up‐regulated by tobamovirus infection in tobacco, which was correlated with and explained a decrease in m^6^A levels (Li et al., 2018). Given the comprehensive identification of RNA demethylases and the large number of resources available, we focused on *A. thaliana* to test if differential expression of AlkB homologues upon virus infection is conserved. Transcriptomes were analysed from seedlings infected with turnip mosaic virus (genus *Potyvirus*), and additional RNA and DNA viruses—turnip crinkle virus (genus *Betacarmovirus*) and cabbage leaf curl virus (genus *Begomovirus*), respectively. Small molecule signalling molecules participate in antiviral responses (Alazem & Lin, 2015; Pasin et al., 2020) and datasets from treatments with phytohormones or their precursors were also considered (Table S7).

Results show that expression of *PATHOGENESIS‐RELATED PROTEIN 1* (*PR‐1*), monitored as a virus‐responsive reference gene, was significantly up‐regulated (*p* < 0.05) by treatments with the three viruses and salicylic acid (Figure 4a, Table S8). Within plant‐specific clades of AlkB homologues, *ALKBH9* genes showed minor transcriptional alterations with log_2_(fold change [FC]) values of −0.40 to 0.32. *ALKBH10B* was significantly up‐regulated by cabbage leaf curl virus and salicylic acid with log_2_(FC) values of 0.896 and 1.087, respectively; conversely, *ALKBH10A* and *ALKBH10C* were significantly down‐regulated by abscisic acid (ABA) and turnip crinkle virus with log_2_(FC) values of −0.613 and −0.639, respectively (Figure 4a, Table S8). In the datasets analysed, turnip mosaic virus showed a nonsignificant impact on any of the *A. thaliana* AlkB homologues with log_2_(FC) values ranging from −0.26 to 0.30 (Figure 4a, Table S8). The results indicate that a transcriptional regulation of plant AlkB homologues by virus infection is minor or not conserved.

**FIGURE 4:**
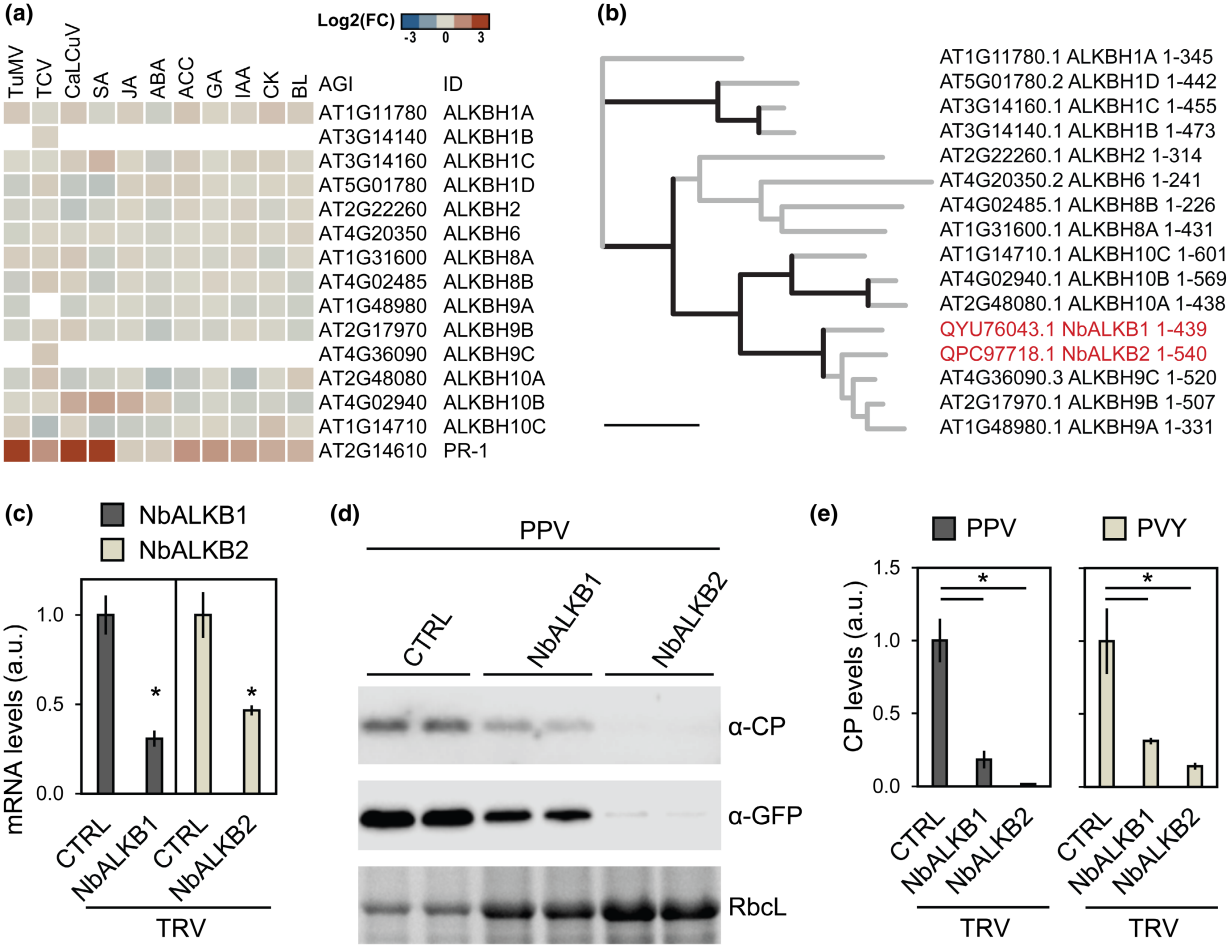
Proviral roles of AlkB homologues in *Potyvirus* infection. (a) Transcriptional regulation of *Arabidopsis thaliana* AlkB homologues by virus infection and phytohormones. Seedlings were infected with turnip mosaic virus (TuMV; *Potyvirus*), turnip crinkle virus (TCV; *Betacarmovirus*), and cabbage leaf curl virus (CaLCuV; *Begomovirus*) or treated with salicylic acid (SA), methyl jasmonate (JA), abscisic acid (ABA), aminocyclopropane‐1‐carboxylic acid (ACC), gibberellic acid (GA), indole‐3‐acetic acid (IAA), zeatin (CK), or brassinolide (BL). Transcript fold changes from transcriptomic datasets are shown (Tables S7 and S8). AlkB homologues were reported (Kawai et al., 2014); *PATHOGENESIS‐RELATED PROTEIN 1* (*PR‐1*) is included as a virus infection‐responsive control gene. (b) Two putative AlkB homologues from *Nicotiana benthamiana* (NbALKB1 and NbALKB2) were amplified from total RNA samples, and the cloned sequences were deposited at GenBank. The encoded proteins (QYU76043, QPC97718) were aligned with *A. thaliana* AlkB homologues (Figure S3), and phylogeny was inferred (Table S9). Tree branches with bootstrap support ≥85 are in black. Scale bar = 1. Protein accession numbers and residue positions are shown; NbALKB1 and NbALKB2 within the ALKBH9 branch are labelled (red). (c) Virus‐induced gene silencing (VIGS) of NbALKB1 and NbALKB2. NbALKB1 and NbALKB2 fragments were inserted into RNA2 of a tobacco rattle virus (TRV) vector system, and endogenous transcript accumulation in TRV‐treated *N. benthamiana* plants was quantified by reverse transcription quantitative PCR. Transcript quantification values are plotted (mean ± standard deviation); *p < 0.05 by Student's *t* test; CTRL, empty vector control. (d,e) Silencing of *N. benthamiana* ALKBH9 homologues reduces accumulation of PPV and PVY. Plants were treated with TRV VIGS constructs targeting *NbALKB1* or *NbALKB2* and then inoculated with PPV or PVY. Samples were collected from upper uninoculated leaves, and potyvirus accumulation was assessed. In (d), immunoblotting results with anti‐PPV coat protein (CP) serum and anti‐GFP antibody are shown; RuBisCO large subunit (RbcL) detected by Ponceau red staining is shown as a loading control. In (e), quantification values from PPV or PVY CP immunoblotting are plotted (mean ± standard deviation); *p < 0.05 by Student's *t* test; CTRL, empty vector control.

Transcriptomic analyses, while informative, are poor predictors of AlkB posttranslational control mechanisms, such as subcellular compartment shuttling and recruitment by viral protein complexes (Alvarado‐Marchena et al., 2021; Martínez‐Pérez et al., 2017; Mielecki et al., 2012). Given the validated m^6^A demethylase activity of ALKBH9 members and their reported involvement in RNA virus infection (Alvarado‐Marchena et al., 2021; Martínez‐Pérez et al., 2017, 2021), we focused on this plant‐specific AlkB clade to further investigate possible RNA methylation roles in potyvirus infection. We amplified two putative AlkB homologues from *N. benthamiana* cDNA samples and named them NbALKB1 and NbALKB2. Protein sequence alignment with *A. thaliana* AlkB homologues shows that NbALKB1 and NbALKB2 cluster with ALKBH9 members (Figures 4b and S3; Table S9). *A. thaliana* ALKBH9B was recently reported to be required for vascular movement of an RNA virus (Martínez‐Pérez et al., 2021). We reasoned that overexpression of the identified *N. benthamiana* ALKBH9 homologues could affect systemic movement of PPV. Binary vectors were obtained for plant overexpression of NbALKB1 and NbALKB2; they were transformed into *Agrobacterium* cells and the selected strains were infiltrated into *N. benthamiana* leaves, which were then mechanically inoculated with PPV. Immunoblotting with PPV CP‐specific serum detected no significant differences in PPV accumulation in local (6 dpi) and upper uninoculated (14 dpi) leaf samples collected from the test and control conditions (Figure S4). The results suggest our assay lacks sensitivity or that endogenous levels of AlkB homologues are already sufficient to sustain a PPV fitness maximum.

Tobacco rattle virus (TRV) vector systems are widely used for virus‐induced gene silencing (VIGS) of plant transcripts (Aragonés et al., 2022) and for functional characterization of plant factors involved in virus pathogenesis and immunity (Liu et al., 2002; Rössner et al., 2022). In an approach complementary to overexpression, *NbALKB1* and *NbALKB2* fragments were inserted in a TRV vector system and delivered to plants to trigger VIGS. At 14 dpi, significant down‐regulation of the endogenous transcripts was confirmed by reverse transcription quantitative PCR assays (Figure 4c); plants were then mechanically inoculated with PPV and samples were collected after 9 days. Immunoblotting of upper uninoculated leaf samples revealed that accumulation of PPV CP and the viral reporter GFP was reduced in plants pretreated with *NbALKB1*‐ and *NbALKB2*‐silencing constructs compared to controls (Figure 4d). Differences in PPV loads were significant as shown by quantification of CP and viral RNA levels (Figures 4e and S5). To further support the results, an independent experiment was done with PVY. Compared to controls, CP immunoblotting showed significantly reduced PVY accumulation in samples collected from plants pretreated with either *NbALKB1*‐ or *NbALKB2*‐silencing constructs (Figure 4e), thus corroborating the PPV results. Taken together, our findings indicate that down‐regulation of *N. benthamiana* ALKBH9 homologues promotes plant resistance to potyvirus infection.

A previous attempt to characterize AlkB roles in potyvirus infection provided no definitive answers as a recombinant potyvirus encoding a plant AlkB homologue did not show any fitness enhancement compared to the parental virus and the exogenous sequence was rapidly lost (Willemsen et al., 2017). Here we nonetheless report identification of AlkB within P1 of the two potyviruses ENMV and FENMV (Figure 1). The finding supports an evolutionary pressure for *Potyviridae* members to acquire domains with predicted involvement in RNA methylation. AlkB homologues of the plant‐specific clade ALKBH9 were recently shown to be required for efficient infection and systemic movement of an RNA virus (Martínez‐Pérez et al., 2017, 2021). Our results indicate that ALKBH9 proviral roles are conserved as high potyvirus accumulation in *N. benthamiana* depended on unaltered transcript levels of ALKBH9 homologues (Figure 4). Collectively our findings are in agreement with the working hypothesis derived from human and, more recently, plant RNA virus models in which m^6^A and internal RNA methylation in general are functionally involved in antiviral immunity (Dang et al., 2019; Martínez‐Pérez et al., 2017, 2021).

AlkB repairs RNA methylation, which was suggested to promote long‐term infection of perennial hosts by safeguarding virus genome integrity (van den Born et al., 2008). It is, however, currently unclear if the AlkB proviral roles herein and previously reported are directly associated with removal of viral RNA methylation or indirectly linked to a transcriptome‐wide epigenetic regulation affecting expression and translation of antiviral components or host factors needed for virus infection. In this regard, global perturbation of plant RNA metabolism by ABA was recently proposed as an antiviral response similar to cellular shut‐off involved in interferon‐ and CRISPR/Cas‐mediated antiviral immunity (Pasin et al., 2020). Interestingly, a functional link connecting ABA and m^6^A was recently reported in strawberry (Zhou et al., 2021). Functional redundancy of *Potyviridae* noncore modules was previously observed (Pasin et al., 2022), and it remains to be determined if, besides AlkB, other potyviral proteins regulate the balance of internal RNA modifications. Of note, HC‐Pro of potyviruses interferes with small RNA 3′ end methylation (Del Toro et al., 2022; Yu et al., 2006) and interacts or colocalizes with RNA decay components that are linked to the activity of plant ALKBH9 homologues (De et al., 2020; Li & Wang, 2018; Martínez‐Pérez et al., 2017). The interplay of RNA methylation and m^6^A with known plant antiviral systems including RNA silencing and decay, small molecule signalling, and resistance gene pathways deserves further investigation.

Profiling of potyvirus genomic and polyprotein variation has identified conserved hypervariable areas possibly involved in host adaptation (Nigam et al., 2019). Future use of novel technological approaches for RNA modification mapping at single‐nucleotide resolution would possibly redefine our understanding of m^6^A and other epigenetic marks in coordinating potyvirus host adaptation and genome evolution.

Finally, innovative agricultural strategies are needed to meet the ever‐growing food and feed demand (Steinwand & Ronald, 2020; Torti et al., 2021). A recent breakthrough study reported that m^6^A demethylase overexpression in two crops caused an approximately 50% yield increase (Yu et al., 2021). Advances in tailored RNA methylation modulation through either plant genome engineering or viral vector delivery may hold potential to spur novel strategies for virus control and epigenetic reprogramming of crop traits.

## CONFLICT OF INTEREST

The authors declare no conflict of interest.

## Supporting information


**Figure S1** Conserved residues in potyvirid AlkB domains. Protein sequences were aligned and residues that participate in catalysis (inverted triangles) or α‐ketoglutarate binding (diamonds) are labelled (van den Born et al., 2008; Yu et al., 2006). Residue positions are indicated; ENMV, endive necrotic mosaic virus; FENMV, French endive necrotic mosaic virus; BlVY, blackberry virus Y; *Escherichia coli* AlkB is included as a standard

**Figure S2** In silico prediction of m^6^A sites in PPV and PVY genomes. (a) Diagram of potyvirus RNA molecules. RNA and encoded polyproteins are represented as lines and arrowed boxes, respectively, and relevant domains are labelled; full‐length polyprotein is shown (I) as well as the truncated polyprotein generated by P3 frameshifting (II). (b) Putative m^6^A peaks predicted by SRAMP in PPV (top) or PVY (bottom) genomes are plotted; dotted lines indicate polyprotein cistrons

**Figure S3** Alignment of plant AlkB homologues related to panel (b) of Figure 4

**Figure S4** Transient expression of *Nicotiana benthamiana* ALKBH9 homologues and PPV accumulation. *Agrobacterium* strains harbouring NbALKB1 or NbALKB2 overexpression constructs were infiltrated into *N. benthamiana* leaves; PPV was then mechanically inoculated. Immunoblotting images show PPV accumulation in samples from locally inoculated leaves (a) or upper uninoculated leaves (b) assessed with PPV anti‐coat protein (CP) serum; RuBisCO large subunit (RbcL) detected by Ponceau red staining is shown as a loading control. In (c), quantification values are plotted (mean ± standard deviation); n.s., *p* > 0.05 by Student’s *t* test; CTRL, empty vector control

**Figure S5** Silencing of *Nicotiana benthamiana* ALKBH9 homologues reduces PPV RNA accumulation. Plants were treated with tobacco rattle virus (TRV) virus‐induced gene silencing (VIGS) constructs targeting *NbALKB1* or *NbALKB2* and then inoculated with plum pox virus (PPV). Samples were collected from upper uninoculated leaves, and PPV RNA levels were measured by reverse transcription quantitative PCR using *NbUBI* (panel A) or *NbPSMD1* (B) for normalization. Quantification values are plotted (mean ± standard deviation); *p < 0.05 by Student’s *t* test; CTRL, empty vector control

**File S1** Supporting methods

**Table S1** GenBank accession numbers of recognized species of *Potyviridae* used for AlkB domain scan

**Table S2** AlkB domains detected in *Potyviridae* members by protein profile scan

**Table S3** Polyprotein sequence variants significantly overrepresented in AlkB‐encoding potyviruses

**Table S4** Viral AlkB phylogenetic tree in Newick format including bootstrap values, species names, (poly)protein accession numbers, and residue positions

**Table S5** m^6^A RNA sequencing samples and statistical results

**Table S6** m^6^A peak enrichment in PPV and PVY genomes

**Table S7** *Arabidopsis thaliana* transcriptomic datasets used for AlkB homologue expression analysis

**Table S8** Fold changes of AlkB homologue expression in the *Arabidopsis thaliana* transcriptomic datasets analysed

**Table S9** Phylogenetic tree of plant AlkB homologues in Newick format including bootstrap values, protein accession numbers, IDs, and residue positions

**Table S10** PCR primers used in the study

## Data Availability

The cloned sequences of *N. benthamiana* AlkB homologues have been deposited at NCBI GenBank at https://www.ncbi.nlm.nih.gov/genbank/ (NbALKB1: accession MZ423212, NbALKB2: accession MT107161). Raw MeRIP‐seq files are available at NCBI under BioProject ID PRJNA820038 (http://www.ncbi.nlm.nih.gov/bioproject/820038), and in the Sequence Read Archive at https://www.ncbi.nlm.nih.gov/sra/ with accession numbers SRR18488193‐SRR18488210.
