# Supplementary material for: AlkB RNA demethylase homologues and N 6 ‐methyladenosine are involved in Potyvirus infection
Source: Mol Plant Pathol. 2022 Jun 14;23(10):1555–64. doi: 10.1111/mpp.13239 (PMC9452765; doi:10.1111/mpp.13239)
Supplement: Supplementary file 1 — Figure S1 Conserved residues in potyvirid AlkB domains. Protein sequences were aligned and residues that participate in catalysis (inverted triangles) or α‐ketoglutarate binding (diamonds) are labelled (van den Born et al., 2008; Yu et al., 2006). Residue positions are indicated; ENMV, endive necrotic mosaic virus; FENMV, French endive necrotic mosaic virus; BlVY, blackberry virus Y; Escherichia coli AlkB is included as a standard [file MPP-23-1555-s016.docx]

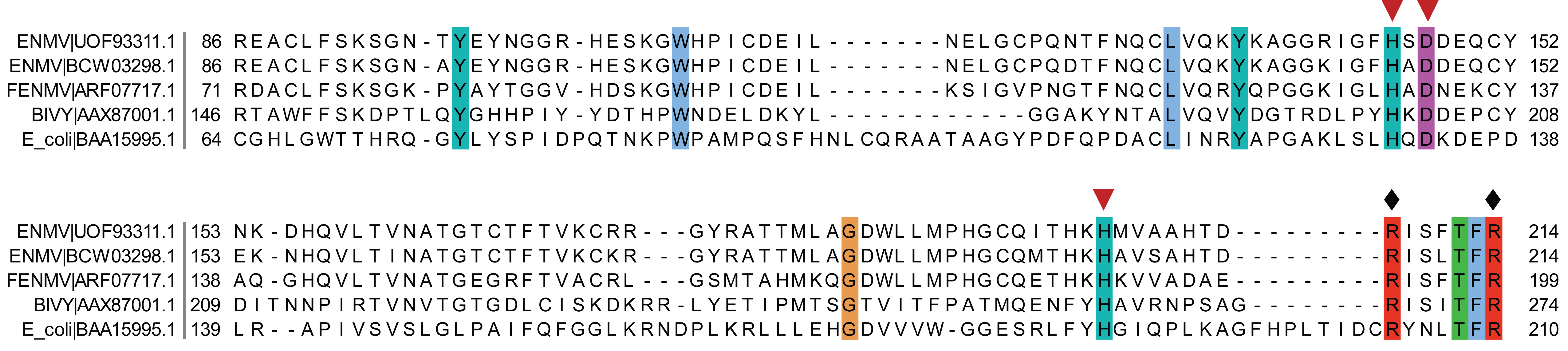


### Figure S1. Conserved residues in potyvirid AlkB domains. Protein sequences were aligned and residues that participate in catalysis (inverted triangles) or α-ketoglutarate binding (diamonds) are labeled (van den Born et al., 2008; Yu et al., 2006). Residue positions are indicated; ENMV, endive necrotic mosaic virus; FENMV, French endive necrotic mosaic virus; BlVY, blackberry virus Y; *E. coli* AlkB is included as a standard.

References

Born, E. van den, Omelchenko, M.V., Bekkelund, A., Leihne, V., Koonin, E.V., Dolja, V.V., et al. (2008) Viral AlkB proteins repair RNA damage by oxidative demethylation. *Nucleic Acids Research*, 36, 5451–5461. <https://doi.org/10.1093/nar/gkn519>.

Yu, B., Edstrom, W.C., Benach, J., Hamuro, Y., Weber, P.C., Gibney, B.R., et al. (2006) Crystal structures of catalytic complexes of the oxidative DNA/RNA repair enzyme AlkB. *Nature*, 439, 879–884. https://doi.org/10.1038/nature04561.
