# Supplementary material for: AlkB RNA demethylase homologues and N 6 ‐methyladenosine are involved in Potyvirus infection
Source: Mol Plant Pathol. 2022 Jun 14;23(10):1555–64. doi: 10.1111/mpp.13239 (PMC9452765; doi:10.1111/mpp.13239)
Supplement: Supplementary file 2 — Figure S2 In silico prediction of m6A sites in PPV and PVY genomes. (a) Diagram of potyvirus RNA molecules. RNA and encoded polyproteins are represented as lines and arrowed boxes, respectively, and relevant domains are labelled; full‐length polyprotein is shown (I) as well as the truncated polyprotein generated by P3 frameshifting (II). (b) Putative m6A peaks predicted by SRAMP in PPV (top) or PVY (bottom) genomes are plotted; dotted lines indicate polyprotein cistrons [file MPP-23-1555-s002.docx]

**
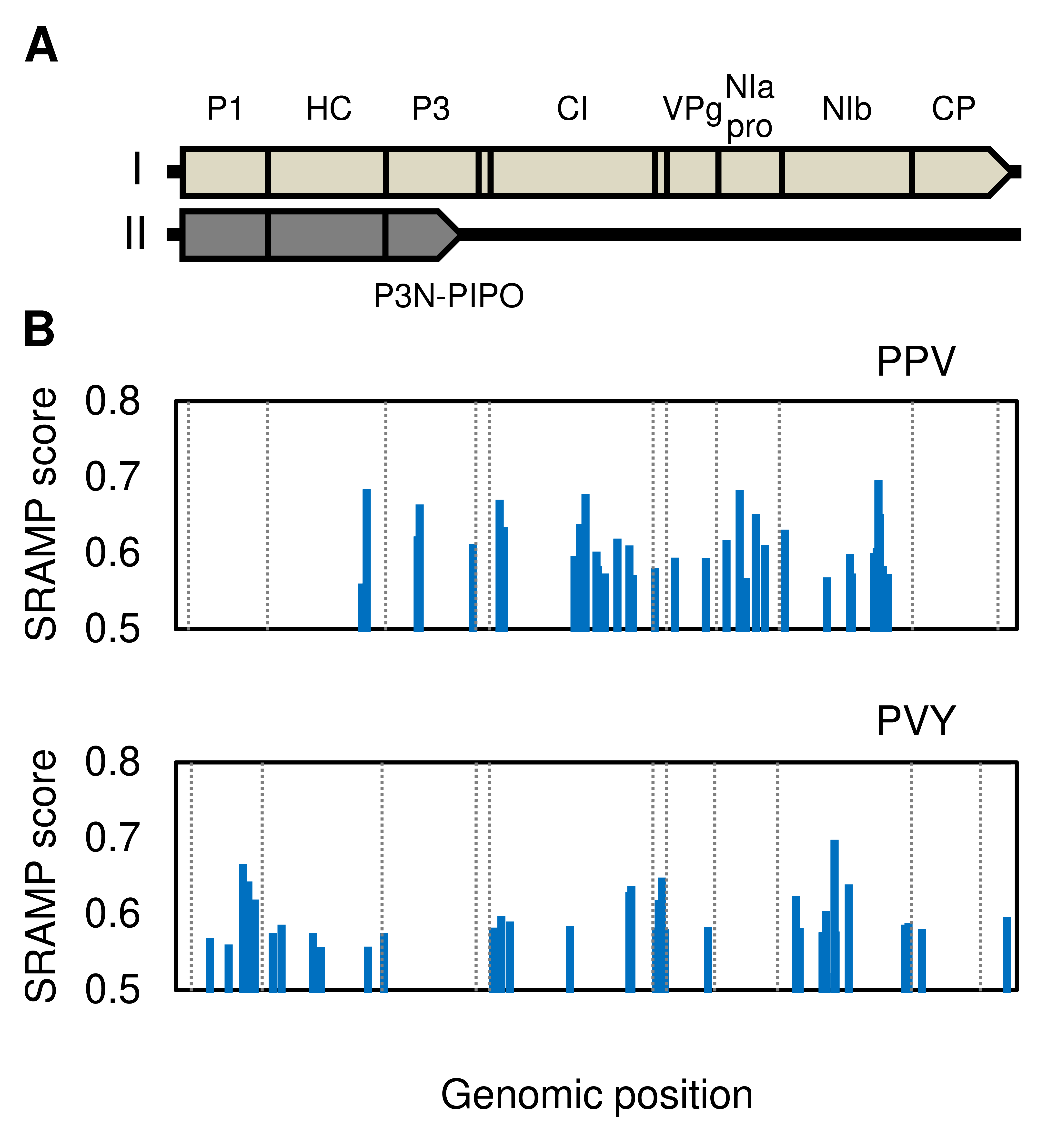
**

Figure S2. *In silico* prediction of m^6^A sites in PPV and PVY genomes. (**A**) Diagram of potyvirus RNA molecules is shown. RNA and encoded polyproteins are represented as lines and arrowed boxes, respectively, and relevant domains are labeled; full-length polyprotein is shown (I) as well as the truncated polyprotein generated by P3 frameshifting (II). (**B**) Putative m^6^A peaks predicted by SRAMP in PPV (top) or PVY (bottom) genomes are plotted; dotted lines indicate polyprotein cistrons.
