# Supplementary material for: AlkB RNA demethylase homologues and N 6 ‐methyladenosine are involved in Potyvirus infection
Source: Mol Plant Pathol. 2022 Jun 14;23(10):1555–64. doi: 10.1111/mpp.13239 (PMC9452765; doi:10.1111/mpp.13239)
Supplement: Supplementary file 4 — Figure S4 Transient expression of Nicotiana benthamiana ALKBH9 homologues and PPV accumulation. Agrobacterium strains harbouring NbALKB1 or NbALKB2 overexpression constructs were infiltrated into N. benthamiana leaves; PPV was then mechanically inoculated. Immunoblotting images show PPV accumulation in samples from locally inoculated leaves (a) or upper uninoculated leaves (b) assessed with PPV anti‐coat protein (CP) serum; RuBisCO large subunit (RbcL) detected by Ponceau red staining is shown as a loading control. In (c), quantification values are plotted (mean ± standard deviation); n.s., p > 0.05 by Student’s t test; CTRL, empty vector control [file MPP-23-1555-s005.docx]

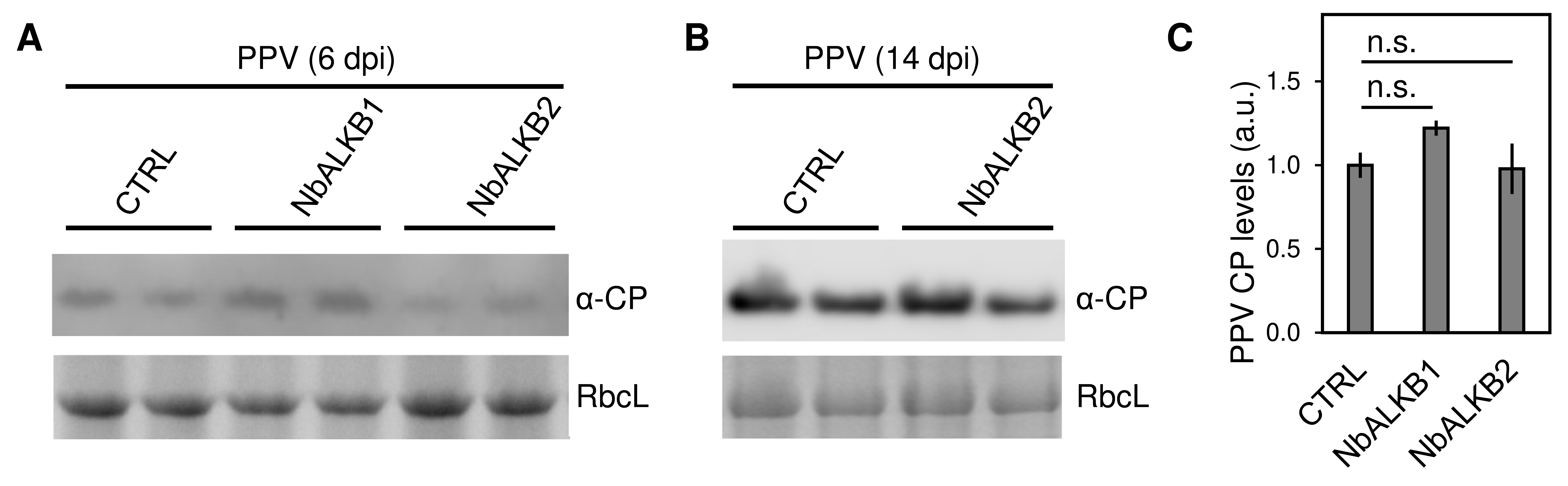


Figure S4. Transient expression of *N. benthamiana* ALKBH9 homologs and PPV accumulation. *Agrobacterium* strains hosting NbALKB1 or NbALKB2 overexpression constructs were infiltrated into *N. benthamiana* leaves; PPV was then mechanically inoculated. Immunoblotting images show PPV accumulation in samples from locally inoculated leaves (A) or upper uninoculated leaves (B) assessed by with PPV anti-coat protein (CP) serum; RuBisCO large subunit (RbcL) detected by Ponceau red-staining is shown as a loading control. In (C), quantification values are plotted (mean ± SD); n.s., *p* > 0.05 by Student’s *t*-test; CTRL, empty vector control.
