# Supplementary material for: AlkB RNA demethylase homologues and N 6 ‐methyladenosine are involved in Potyvirus infection
Source: Mol Plant Pathol. 2022 Jun 14;23(10):1555–64. doi: 10.1111/mpp.13239 (PMC9452765; doi:10.1111/mpp.13239)
Supplement: Supplementary file 5 — Figure S5 Silencing of Nicotiana benthamiana ALKBH9 homologues reduces PPV RNA accumulation. Plants were treated with tobacco rattle virus (TRV) virus‐induced gene silencing (VIGS) constructs targeting NbALKB1 or NbALKB2 and then inoculated with plum pox virus (PPV). Samples were collected from upper uninoculated leaves, and PPV RNA levels were measured by reverse transcription quantitative PCR using NbUBI (panel A) or NbPSMD1 (B) for normalization. Quantification values are plotted (mean ± standard deviation); *p < 0.05 by Student’s t test; CTRL, empty vector control [file MPP-23-1555-s013.docx]

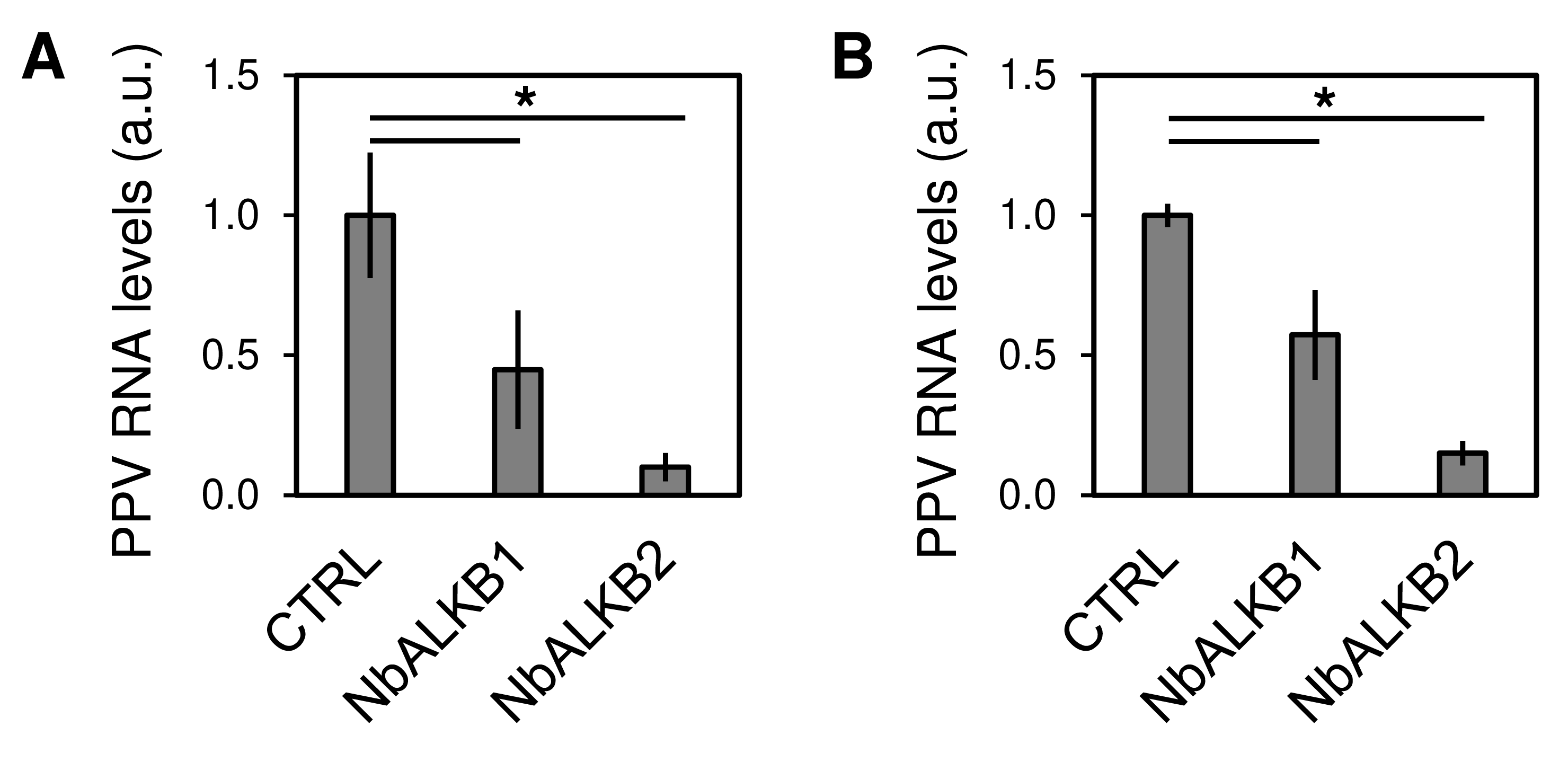


Figure S5. Silencing of *N. benthamiana* ALKBH9 homologs reduces PPV RNA accumulation. Plants were treated with TRV VIGS constructs targeting NbALKB1 or NbALKB2, and then inoculated with PPV. Samples were collected from upper uninoculated leaves, and PPV RNA levels were measured by RT-qPCR using *NbUBI* (panel A), or *NbPSMD1* (B) for normalization. Quantification values are plotted (mean ± SD); *, *p* < 0.05 by Student’s *t*-test; CTRL, empty vector control.
