# Supplementary material for: AlkB RNA demethylase homologues and N 6 ‐methyladenosine are involved in Potyvirus infection
Source: Mol Plant Pathol. 2022 Jun 14;23(10):1555–64. doi: 10.1111/mpp.13239 (PMC9452765; doi:10.1111/mpp.13239)
Supplement: Supplementary file 6 — File S1 Supporting methods [file MPP-23-1555-s011.docx]

## SUPPORTING METHODS

### Sequence analysis

*Potyviridae* reference isolates of recognized species were obtained from International Committee on Taxonomy of Viruses (2020); complete or near-complete genomic sequences (Table S1) were analyzed by InterProScan in the nucleotide mode (Blum et al., 2021). *In silico* *N*^6^-methyladenosine (m^6^A) site prediction in plum pox virus (PPV; AJ243957) and potato virus Y (PVY; EF026075) genomes was done using SRAMP (Zhou et al., 2016). Viral protein sequences were obtained from NCBI (NCBI Resource Coordinators, 2018); *Arabidopsis thaliana* AlkB homologs were reported (Kawai et al., 2014), and protein sequences were obtained from TAIR (Lamesch et al., 2012). Protein sequences were aligned using MAFFT with the G-INS-i refinement method (Katoh et al., 2019). Phylogeny was inferred using IQ-TREE (Trifinopoulos et al., 2016), with ModelFinder together with ultrafast bootstrap (1000×) and SH-aLRT supports (1000×); root was inferred using simple midpoint with TreeRate (Maljkovic Berry et al., 2007). Trees were visualized using iTOL (Letunic and Bork, 2021).

Pairwise sequence comparisons and identity scores for species demarcation were computed using SDT and the MAFFT alignment option (Muhire et al., 2014). To analyze HC-pro length variation within the *Potyvirus* genus, polyproteins of reference isolates were aligned alongside endive necrotic mosaic virus (ENMV) and French ENMV (FENMV); P1 and HC-pro cleavage sites were identified as described (Pasin et al., 2022), and sequences of mature HC-pro were analyzed. To analyze variants within the *Potyvirus* genus, polyproteins of reference isolates were aligned alongside F/ENMV isolates (Table S3); JalView (Waterhouse et al., 2009) was used to export the genus consensus and the F/ENMV consensus. Sites with 100% F/ENMV conservation [5/5 isolates] and ≥ 80% genus conservation were compared. Those showing divergent consensus residues were retained and filtered to remove F/ENMV and vanilla distortion mosaic virus (VDMV) conservation.

### Plant materials and virus inoculation

Wild-type *Nicotiana benthamiana* plants were grown in a greenhouse at ~23°C with a 16 h light/8 h dark photoperiod. Binary T-DNA vectors with infectious cDNA clones of a plum pox virus (PPV) isolate adapted to *Nicotiana* spp. and potato virus Y (PVY) were described (Cordero et al., 2017; Pasin et al., 2017). Clones were delivered to *N. benthamiana* plants by *Agrobacterium*-mediated inoculation as detailed (Zhao et al., 2020). Upper uninoculated leaves were collected, stored at -80°C and used as the inoculum source in the described infection assays; PPV and PVY inoculum preparation in phosphate buffer and mechanical inoculation were done as described (Pasin et al., 2020).

### m^6^A quantification

Total RNA was purified by Trizol (Tiangen, China) from samples collected from healthy plants or upper uninoculated leaves of PPV- and PVY-treated plants. m^6^A in RNA samples was quantified using a colorimetric ELISA-like assay (EpiQuik m^6^A RNA methylation quantification kit, #P-9005; Epigentek, USA).

### MeRIP-seq

Total RNA was purified as described above, analyzed in a Bioanalyzer 2100 with the RNA 6000 Nano kit (Agilent, USA), and poly(A) containing RNA was enriched using oligo(dT)-coupled magnetic beads (Invitrogen, USA). The recovered RNA was fragmented using divalent cations under elevated temperature. RNA fragments were incubated 2 h at 4°C with an m^6^A-specific antibody (#202003; Synaptic Systems, Germany) in IP buffer (50 mM Tris-HCl, 750 mM NaCl and 0.5% Igepal CA-630) supplemented with BSA (0.5 μg/μL). The mixture was then incubated with protein-A beads, eluted in 1× IP buffer supplemented with 6.7 mM m^6^A, and precipitated by 75% ethanol. Sequencing libraries were prepared from the recovered immunoprecipitated m^6^A-containing RNA fragments and untreated input control RNA samples by a dUTP-based strand-specific library preparation method. Libraries were sequenced (2 × 150-nt paired-end reads) on an Illumina Novaseq™ 6000 platform at the LC-BIO Bio-tech ltd (Hangzhou, China) to recover > 3 × 10^7^ raw reads/sample (Table S5). Raw reads were processed with Cutadapt (Martin, 2011) and in-house Perl scripts to remove adaptor contamination, low quality or undetermined bases; read quality was then verified using FastQC (Andrews, 2010). Clean reads were mapped the *N. benthamiana* genome dataset Niben.genome.v1.0.1.scaffolds.nrcontigs.fasta.gz (Bombarely et al., 2011), or PPV and PVY genomes (EF026075) with HISAT2 (Kim et al., 2015) and default parameters. Mapped reads of IP and input control libraries were analyzed with exomePeak (Meng et al., 2014) to identify m^6^A peaks. Called peaks were annotated by intersection with gene architecture using ChIPseeker (Yu et al., 2015).

### Gene cloning, transient expression and virus-induced gene silencing (VIGS) assays

The plant AlkB homologs NbALKB1 and NbALKB2 were amplified from *N. benthamiana* cDNA samples using the gene-specific primer pairs NbALKB1_F/NbALKB1_R and NbALKB2_F/NbALKB2_R, respectively (Table S10). The obtained products were cloned into the pMD19-T vector, and verified by Sanger sequencing. The pMD19-T derivatives were used in Gateway recombinase reactions (Invitrogen, USA) alongside pEAQ-HT-DEST1 (Peyret and Lomonossoff, 2013) to obtain binary vectors for plant overexpression. A tobacco rattle virus (TRV) system comprising pTRV1 and pTRV2 (Liu et al., 2002) was used to silence endogenous transcripts. For VIGS vector construction, ~200-bp fragments of NbALKB1 and NbALKB2 were amplified from pMD19-T derivatives using the primer pairs NbALKB1‑TRV2_F/NbALKB1‑TRV2_R and NbALKB2-TRV2_F/NbALKB2-TRV2_R, respectively (Table S10). The fragments were inserted into pTRV2 by restriction enzyme digestion and DNA ligation. The resulting constructs were transformed by the freeze-thaw method into *Agrobacterium* strain C58C1 hosting a disarmed pTi. For plant transient expression and VIGS assays, the recovered *Agrobacterium* strains were cultured, induced and syringe-infiltrated into *N. benthamiana* leaves as described (Pasin et al., 2020). To assess VIGS efficiency, samples were collected at 14 days post-inoculation (dpi). Total RNA was purified and used in cDNA synthesis reactions; cDNA aliquots were then used in PCR reactions performed with MonAmp^TM^ SYBR Green qPCR mix (Monad, China) and gene- or virus-specific primers (Table S10) in an FTC-3000P Real-Time Quantitative Thermal Cycler (Funglyn Biotech, Canada). Host transcript and viral RNA levels were normalized using *NbUBI* or *NbPSMD1* as a reference (Lacomme et al., 2003; Pasin et al., 2020), and fold changes relative to the control condition were calculated by the ΔΔ^CT^ method (Rodamilans et al., 2014).

### Protein immunodetection

Total protein extracts from plant samples were prepared, resolved by SDS-PAGE and electroblotted onto nitrocellulose membranes as described (Pasin et al., 2014). Membranes were stained with Ponceau S for loading control; immunodetection was conducted using as the primary antibodies rabbit anti-PPV coat protein (CP) (Pasin et al., 2020), and anti-PVY CP (WG-03590; ABclonal, China) sera, and mouse anti-GFP monoclonal antibody (AE012; ABclonal, China). Horseradish peroxidase-conjugated goat anti-rabbit IgG (ab205718; Abcam, USA) or goat anti-mouse IgG (AS003; ABclonal, China) were used as the secondary antibodies and immunostained proteins were visualized by enhanced chemiluminescence detection.

### Statistics

A two-tailed Student’s *t* test was used for two-group comparisons; significance levels of *p* values are indicated in the figures. Overrepresentation significance of polyprotein sequence variants was determined by the hypergeometric test (upper cumulative distribution).

## REFERENCES

Andrews, S. (2010) FastQC: a quality control tool for high throughput sequence data. Babraham Bioinformatics, Babraham Institute, Cambridge, United Kingdom.

Ascencio-Ibáñez, J.T., Sozzani, R., Lee, T.-J., Chu, T.-M., Wolfinger, R.D., Cella, R., et al. (2008) Global analysis of Arabidopsis gene expression uncovers a complex array of changes impacting pathogen response and cell cycle during geminivirus infection. *Plant Physiology*, 148, 436–454. https://doi.org/10.1104/pp.108.121038.

Blum, M., Chang, H.-Y., Chuguransky, S., Grego, T., Kandasaamy, S., Mitchell, A., et al. (2021) The InterPro protein families and domains database: 20 years on. *Nucleic Acids Research*, 49, D344–D354. https://doi.org/10.1093/nar/gkaa977.

Bombarely, A., Menda, N., Tecle, I.Y., Buels, R.M., Strickler, S., Fischer-York, T., et al. (2011) The Sol Genomics Network (solgenomics.net): growing tomatoes using Perl. *Nucleic acids research*, 39, D1149–D1155. https://doi.org/10.1093/nar/gkq866.

Born, E. van den, Omelchenko, M.V., Bekkelund, A., Leihne, V., Koonin, E.V., Dolja, V.V., et al. (2008) Viral AlkB proteins repair RNA damage by oxidative demethylation. *Nucleic Acids Research*, 36, 5451–5461. https://doi.org/10.1093/nar/gkn519.

Cordero, T., Mohamed, M.A., López-Moya, J.-J. & Daròs, J.-A. (2017) A recombinant *Potato virus Y* infectious clone tagged with the Rosea1 visual marker (PVY-Ros1) facilitates the analysis of viral infectivity and allows the production of large amounts of anthocyanins in plants. *Frontiers in Microbiology*, 8, 611. https://doi.org/10.3389/fmicb.2017.00611.

Hruz, T., Laule, O., Szabo, G., Wessendorp, F., Bleuler, S., Oertle, L., et al. (2008) Genevestigator v3: a reference expression database for the meta-analysis of transcriptomes. *Advances in Bioinformatics*, 2008, 420747. https://doi.org/10.1155/2008/420747.

International Committee on Taxonomy of Viruses, 2020 ICTV master species list 2019.v1 (MSL #35). Last accessed June 2021, <https://talk.ictvonline.org/files/master-species-lists/m/msl/9601>.

Katoh, K., Rozewicki, J. & Yamada, K.D. (2019) MAFFT online service: multiple sequence alignment, interactive sequence choice and visualization. *Briefings in Bioinformatics*, 20, 1160–1166. https://doi.org/10.1093/bib/bbx108.

Kawai, Y., Ono, E. & Mizutani, M. (2014) Evolution and diversity of the 2-oxoglutarate-dependent dioxygenase superfamily in plants. *The Plant Journal*, 78, 328–343. https://doi.org/10.1111/tpj.12479.

Kim, D., Langmead, B. & Salzberg, S.L. (2015) HISAT: a fast spliced aligner with low memory requirements. *Nature Methods*, 12, 357–360. https://doi.org/10.1038/nmeth.3317.

Lacomme, C., Hrubikova, K. & Hein, I. (2003) Enhancement of virus-induced gene silencing through viral-based production of inverted-repeats. *The Plant Journal*, 34, 543–553. https://doi.org/10.1046/j.1365-313x.2003.01733.x.

Lamesch, P., Berardini, T.Z., Li, D., Swarbreck, D., Wilks, C., Sasidharan, R., et al. (2012) The Arabidopsis Information Resource (TAIR): improved gene annotation and new tools. *Nucleic Acids Research*, 40, D1202-1210. https://doi.org/10.1093/nar/gkr1090.

Letunic, I. & Bork, P. (2021) Interactive Tree Of Life (iTOL) v5: an online tool for phylogenetic tree display and annotation. *Nucleic Acids Research*, gkab301. https://doi.org/10.1093/nar/gkab301.

Liu, Y., Schiff, M., Marathe, R. & Dinesh-Kumar, S.P. (2002) Tobacco *Rar1*, *EDS1* and *NPR1/NIM1* like genes are required for *N*-mediated resistance to tobacco mosaic virus. *The Plant Journal*, 30, 415–429. https://doi.org/10.1046/j.1365-313x.2002.01297.x.

Maljkovic Berry, I., Ribeiro, R., Kothari, M., Athreya, G., Daniels, M., Lee, H.Y., et al. (2007) Unequal evolutionary rates in the human immunodeficiency virus type 1 (HIV-1) pandemic: the evolutionary rate of HIV-1 slows down when the epidemic rate increases. *Journal of Virology*, 81, 10625–10635. https://doi.org/10.1128/jvi.00985-07.

Martin, M. (2011) Cutadapt removes adapter sequences from high-throughput sequencing reads. *EMBnet journal*, 17, 10. https://doi.org/10.14806/ej.17.1.200.

Meng, J., Lu, Z., Liu, H., Zhang, L., Zhang, S., Chen, Y., et al. (2014) A protocol for RNA methylation differential analysis with MeRIP-Seq data and exomePeak R/Bioconductor package. *Methods (San Diego, Calif.)*, 69, 274–281. https://doi.org/10.1016/j.ymeth.2014.06.008.

Muhire, B.M., Varsani, A. & Martin, D.P. (2014) SDT: a virus classification tool based on pairwise sequence alignment and identity calculation. *PloS One*, 9, e108277. https://doi.org/10.1371/journal.pone.0108277.

NCBI Resource Coordinators (2018) Database resources of the National Center for Biotechnology Information. *Nucleic Acids Research*, 46, D8–D13. https://doi.org/10.1093/nar/gkx1095.

Nemhauser, J.L., Hong, F. & Chory, J. (2006) Different plant hormones regulate similar processes through largely nonoverlapping transcriptional responses. *Cell*, 126, 467–475. https://doi.org/10.1016/j.cell.2006.05.050.

Pasin, F., Bedoya, L.C., Bernabé-Orts, J.M., Gallo, A., Simón-Mateo, C., Orzaez, D., et al. (2017) Multiple T-DNA delivery to plants using novel mini binary vectors with compatible replication origins. *ACS synthetic biology*, 6, 1962–1968. https://doi.org/10.1021/acssynbio.6b00354.

Pasin, F., Daròs, J.-A. & Tzanetakis, I.E. (2022) Proteome expansion in the *Potyviridae* evolutionary radiation. *FEMS Microbiology Reviews*, fuac011. https://doi.org/10.1093/femsre/fuac011.

Pasin, F., Shan, H., García, B., Müller, M., San León, D., Ludman, M., et al. (2020) Abscisic acid connects phytohormone signaling with RNA metabolic pathways and promotes an antiviral response that is evaded by a self-controlled RNA virus. *Plant Communications*, 1, 100099. https://doi.org/10.1016/j.xplc.2020.100099.

Pasin, F., Simón-Mateo, C. & García, J.A. (2014) The hypervariable amino-terminus of P1 protease modulates potyviral replication and host defense responses. *PLOS Pathogens*, 10, e1003985. https://doi.org/10.1371/journal.ppat.1003985.

Peyret, H. & Lomonossoff, G.P. (2013) The pEAQ vector series: the easy and quick way to produce recombinant proteins in plants. *Plant molecular biology*, 1–8.

Rodamilans, B., San León, D., Mühlberger, L., Candresse, T., Neumüller, M., Oliveros, J.C., et al. (2014) Transcriptomic analysis of *Prunus domestica* undergoing hypersensitive response to *Plum pox virus* infection. *PloS One*, 9, e100477. https://doi.org/10.1371/journal.pone.0100477.

Trifinopoulos, J., Nguyen, L.-T., von Haeseler, A. & Minh, B.Q. (2016) W-IQ-TREE: a fast online phylogenetic tool for maximum likelihood analysis. *Nucleic Acids Research*, 44, W232–W235. https://doi.org/10.1093/nar/gkw256.

Waterhouse, A.M., Procter, J.B., Martin, D.M.A., Clamp, M. & Barton, G.J. (2009) Jalview version 2—a multiple sequence alignment editor and analysis workbench. *Bioinformatics*, 25, 1189–1191. https://doi.org/10.1093/bioinformatics/btp033.

Wu, C., Li, X., Guo, S. & Wong, S.-M. (2016) Analyses of RNA-Seq and sRNA-Seq data reveal a complex network of anti-viral defense in TCV-infected *Arabidopsis thaliana*. *Scientific Reports*, 6, 36007. https://doi.org/10.1038/srep36007.

Yang, C., Guo, R., Jie, F., Nettleton, D., Peng, J., Carr, T., et al. (2007) Spatial analysis of *Arabidopsis thaliana* gene expression in response to *Turnip mosaic virus* infection. *Molecular plant-microbe interactions*, 20, 358–370. https://doi.org/10.1094/mpmi-20-4-0358.

Yu, B., Edstrom, W.C., Benach, J., Hamuro, Y., Weber, P.C., Gibney, B.R., et al. (2006) Crystal structures of catalytic complexes of the oxidative DNA/RNA repair enzyme AlkB. *Nature*, 439, 879–884. https://doi.org/10.1038/nature04561.

Yu, G., Wang, L.-G. & He, Q.-Y. (2015) ChIPseeker: an R/Bioconductor package for ChIP peak annotation, comparison and visualization. *Bioinformatics*, 31, 2382–2383. https://doi.org/10.1093/bioinformatics/btv145.

Zhao, M., García, B., Gallo, A., Tzanetakis, I.E., Simón-Mateo, C., García, J.A., et al. (2020) Home-made enzymatic premix and Illumina sequencing allow for one-step Gibson assembly and verification of virus infectious clones. *Phytopathology Research*, 2, 36. https://doi.org/10.1186/s42483-020-00077-4.

Zhou, Y., Zeng, P., Li, Y.-H., Zhang, Z. & Cui, Q. (2016) SRAMP: prediction of mammalian N^6^-methyladenosine (m^6^A) sites based on sequence-derived features. *Nucleic Acids Research*, 44, e91. https://doi.org/10.1093/nar/gkw104.
