# Supplementary material for: AlkB RNA demethylase homologues and N 6 ‐methyladenosine are involved in Potyvirus infection
Source: Mol Plant Pathol. 2022 Jun 14;23(10):1555–64. doi: 10.1111/mpp.13239 (PMC9452765; doi:10.1111/mpp.13239)
Supplement: Supplementary file 7 — Table S1 GenBank accession numbers of recognized species of Potyviridae used for AlkB domain scan [file MPP-23-1555-s003.docx]

### Table S1. GenBank accession numbers of recognized species of *Potyviridae* used for AlkB domain scan

| Genus | Genome_accessions |
| --- | --- |
| *Arepavirus* | MH395371 MH330686 |
| *Bevemovirus* | KY491536 |
| *Brambyvirus* | AY994084 |
| *Bymovirus* | X90904 Y10973 AJ132268 AJ132269 AJ306719 AJ306718 LC055681 LC060925 MN046367 MN046369 D86634 D86635 |
| *Celavirus* | MH932227 |
| *Ipomovirus* | GU563327 KU935732 AY578085 EU259611 Z73124 HE600072 FJ185044 |
| *Macluravirus* | MG978107 KP405232 KU053507 MF622947 AB710145 KX979913 KT724961 MG755240 |
| *Poacevirus* | JX156425 GQ388116 FJ263671 |
| *Potyvirus* | MF997470 EU410442 MK440140 MN709786 HM363516 DQ925486 KJ830760 DQ851496 KU685505 DQ821938 U19287 AJ312437 D83749 AY206394 KF649336 AF538686 JQ807999 JX867236 AB551370 EF105299 KX013584 GQ421689 JX156434 LC511903 KP343681 HQ676607 JN008909 AJ237843 AB011819 AF499738 JQ801448 AF348210 KY657266 JQ723475 DQ299908 KU556609 AJ298033 MK241979 MH206616 JX156422 LC379162 AB246773 KU941946 KX355613 FM206346 AM039800 EF427894 LC228573 AB818538 HQ161081 MH779476 JQ395040 KY828925 KU981084 MH886513 KT692938 AB027007 KT222674 Z26920 KY385304 JF838187 AB219545 AJ307057 KP769852 X97705 AJ564636 MF543013 EU847625 AJ001691 MN116683 MH680824 MF953305 EF579955 AM182028 KC691259 AM158908 MT396083 AJ510223 JQ807997 MW035311 GQ916624 BD171712 X67673 MF509898 HQ122652 D10930 AF023848 KT633868 AY642590 M96425 AM181350 DQ645484 AB541985 AJ437280 MH779625 AJ243957 JX291161 AJ296311 AJ243766 U09509 JX294310 KY562565 AJ316084 AJ865076 AJ310197 D00507 KY623505 AJ297628 GU181199 JQ350738 KX856009 D86371 KC443039 JN613807 GU207957 JQ824374 KM523548 DQ851493 AJ851866 M11458 KT834407 EF219408 X04083 JQ314463 MH886517 AF169561 KF906523 EU564817 AY437609 KY623506 LC159494 AJ437279 DQ851495 AY656816 JX470965 U42596 JN190431 AY626825 JQ692088 KU355553 KC345607 AF127929 |
| *Roymovirus* | MH379332 JF280796 |
| *Rymovirus* | AY623626 AY623627 Y09854 |
| *Tritimovirus* | Z48506 AY377938 KF260962 EF608612 AF057533 KF984546 |
| Unassigned | KY612317 KY649478 MN788417 |
