# Supplementary material for: AlkB RNA demethylase homologues and N 6 ‐methyladenosine are involved in Potyvirus infection
Source: Mol Plant Pathol. 2022 Jun 14;23(10):1555–64. doi: 10.1111/mpp.13239 (PMC9452765; doi:10.1111/mpp.13239)
Supplement: Supplementary file 8 — Table S2 AlkB domains detected in Potyviridae members by protein profile scan [file MPP-23-1555-s006.docx]

### Table S2. AlkB domains detected in *Potyviridae* members by protein profile scan

| Polyprotein | | | Protein profile ^a^ | | | | | |
| --- | --- | --- | --- | --- | --- | --- | --- | --- |
| Virus ^b^ | Acc. no. | Length | Database | ID | Start | End | Score | Description |
| BlVY | AAX87001 | 3491 | Gene3D | G3DSA:2.60.120.590 | 118 | 276 | 3.30E-14 | Alpha-ketoglutarate-dependent dioxygenase AlkB-like superfamily |
| BlVY | AAX87001 | 3491 | SUPERFAMILY | SSF51197 | 143 | 275 | 1.13E-17 | Clavaminate synthase-like |
| BlVY | AAX87001 | 3491 | ProSiteProfiles | PS51471 | 183 | 277 | 8.88 | Oxoglutarate/iron-dependent dioxygenase |
| BlVY | AAX87001 | 3491 | SUPERFAMILY | SSF56672 | 2620 | 3073 | 8.90E-108 | DNA/RNA polymerase superfamily |
| ENMV | BCW03298 | 3240 | Gene3D | G3DSA:2.60.120.590 | 76 | 216 | 8.60E-22 | Alpha-ketoglutarate-dependent dioxygenase AlkB-like superfamily |
| ENMV | BCW03298 | 3240 | SUPERFAMILY | SSF51197 | 83 | 215 | 3.85E-24 | Clavaminate synthase-like |
| ENMV | BCW03298 | 3240 | ProSiteProfiles | PS51471 | 127 | 217 | 9.71 | Oxoglutarate/iron-dependent dioxygenase |
| ENMV | BCW03298 | 3240 | SUPERFAMILY | SSF56672 | 2482 | 2940 | 1.29E-138 | DNA/RNA polymerase superfamily |
| ENMV | BCW03299 | 3240 | Gene3D | G3DSA:2.60.120.590 | 76 | 216 | 8.60E-22 | Alpha-ketoglutarate-dependent dioxygenase AlkB-like superfamily |
| ENMV | BCW03299 | 3240 | SUPERFAMILY | SSF51197 | 83 | 215 | 3.85E-24 | Clavaminate synthase-like |
| ENMV | BCW03299 | 3240 | ProSiteProfiles | PS51471 | 127 | 217 | 9.71 | Oxoglutarate/iron-dependent dioxygenase |
| ENMV | BCW03299 | 3240 | SUPERFAMILY | SSF56672 | 2482 | 2940 | 4.89E-138 | DNA/RNA polymerase superfamily |
| ENMV | UOF93311 | 3240 | Gene3D | G3DSA:2.60.120.590 | 76 | 216 | 5.40E-20 | Alpha-ketoglutarate-dependent dioxygenase AlkB-like superfamily |
| ENMV | UOF93311 | 3240 | SUPERFAMILY | SSF51197 | 83 | 215 | 9.89E-23 | Clavaminate synthase-like |
| ENMV | UOF93311 | 3240 | ProSiteProfiles | PS51471 | 127 | 217 | 9.37 | Oxoglutarate/iron-dependent dioxygenase |
| ENMV | UOF93311 | 3240 | SUPERFAMILY | SSF56672 | 2482 | 2940 | 1.68E-137 | DNA/RNA polymerase superfamily |
| ENMV | UOF93331 | 3240 | Gene3D | G3DSA:2.60.120.590 | 76 | 216 | 5.40E-20 | Alpha-ketoglutarate-dependent dioxygenase AlkB-like superfamily |
| ENMV | UOF93331 | 3240 | SUPERFAMILY | SSF51197 | 83 | 215 | 9.89E-23 | Clavaminate synthase-like |
| ENMV | UOF93331 | 3240 | ProSiteProfiles | PS51471 | 127 | 217 | 9.37 | Oxoglutarate/iron-dependent dioxygenase |
| ENMV | UOF93331 | 3240 | SUPERFAMILY | SSF56672 | 2482 | 2940 | 1.68E-137 | DNA/RNA polymerase superfamily |
| FENMV | ARF07717 | 3236 | Gene3D | G3DSA:2.60.120.590 | 60 | 201 | 8.50E-20 | Alpha-ketoglutarate-dependent dioxygenase AlkB-like superfamily |
| FENMV | ARF07717 | 3236 | SUPERFAMILY | SSF51197 | 61 | 200 | 3.71E-22 | Clavaminate synthase-like |
| FENMV | ARF07717 | 3236 | ProSiteProfiles | PS51471 | 112 | 202 | 10.21 | Oxoglutarate/iron-dependent dioxygenase |
| FENMV | ARF07717 | 3236 | SUPERFAMILY | SSF56672 | 2477 | 2935 | 8.90E-137 | DNA/RNA polymerase superfamily |
| VDMV | AHU88030 | 3083 | SUPERFAMILY | SSF56672 | 2315 | 2773 | 4.84E-135 | DNA/RNA polymerase superfamily |
| ^a^ G3DSA:2.60.120.590, SSF51197, PS51471 are AlkB superfamily profiles; SSF56672 is included as a positive control of protein domain search.  ^b^ BlVY, blackberry virus Y; ENMV, endive necrotic mosaic virus; FENMV, French endive necrotic mosaic virus; VDMV, vanilla distortion mosaic virus. | | | | | | | | |
