# Supplementary material for: AlkB RNA demethylase homologues and N 6 ‐methyladenosine are involved in Potyvirus infection
Source: Mol Plant Pathol. 2022 Jun 14;23(10):1555–64. doi: 10.1111/mpp.13239 (PMC9452765; doi:10.1111/mpp.13239)
Supplement: Supplementary file 9 — Table S3 Polyprotein sequence variants significantly overrepresented in AlkB‐encoding potyviruses [file MPP-23-1555-s009.docx]

### Table S3. Polyprotein sequence variants significantly overrepresented in AlkB-encoding potyviruses

| Polyprotein | | | aa frequency | | Hypergeometric distribution | | | | |
| --- | --- | --- | --- | --- | --- | --- | --- | --- | --- |
| Gene | Position ^a^ | aa | AlkB presence | AlkB absence | event_F/ENMV ^b^ | n_F/ENMV ^b^ | event_genus ^c^ | n_genus ^c^ | Q value |
| HC-pro | 691 | Y | 100% | 7.97% | 5 | 5 | 16 | 143 ^d^ | 9.41E-06 |
| HC-pro | 807 | A | 100% | 0.00% | 5 | 5 | 5 | 144 | 2.08E-09 |
| CI | 1441 | I | 100% | 0.00% | 5 | 5 | 5 | 144 | 2.08E-09 |
| CI | 1460 | I | 100% | 1.44% | 5 | 5 | 7 | 144 | 4.37E-08 |
| CI | 1527 | T | 100% | 4.32% | 5 | 5 | 11 | 144 | 9.60E-07 |
| CI | 1590 | A | 100% | 11.51% | 5 | 5 | 21 | 144 | 4.23E-05 |
| CI | 1776 | K | 100% | 3.60% | 5 | 5 | 10 | 144 | 2.62E-07 |
| CI | 1883 | Q | 100% | 0.00% | 5 | 5 | 5 | 144 | 2.08E-09 |
| VPg | 2063 | R | 100% | 8.63% | 5 | 5 | 17 | 144 | 9.08E-06 |
| NIa-pro | 2239 | G | 100% | 0.72% | 5 | 5 | 6 | 144 | 2.08E-09 |
| NIb | 2487 | Q | 100% | 0.72% | 5 | 5 | 6 | 144 | 2.08E-09 |
| CP | 3089 | V | 100% | 2.16% | 5 | 5 | 8 | 144 | 4.37E-08 |
| CP | 3117 | A | 100% | 1.45% | 5 | 5 | 7 | 143 ^d^ | 1.29E-08 |
| ^a^ Numbers refer to ENMV accession no. UOF93311  ^b^ Accession nos. UOF93311, UOF93331, BCW03298, BCW03299, ARF07717  ^c^ Accession nos. UOF93311, UOF93331, BCW03298, BCW03299, ARF07717 plus AAA46903, AAA47909, AAB02170, AAB50573, AAB94595, AAD44684, AAF89676, AAL83896, AAM19343, AAO37457, AAP41071, AAR99062, AAV48572, AAV54595, AAV68594, ABC25565, ABG56784, ABH10134, ABI34612, ABI34614, ABI34615, ABI97034, ABO77135, ABP87907, ABR88099, ABU93572, ACB69755, ACB88930, ACJ31798, ACV84257, ADF31931, ADI58756, ADQ74918, ADR10439, ADT71770, AEB00568, AEB00584, AEP37329, AER59752, AEV45179, AFI25344, AFJ68041, AFJ92905, AFJ92921, AFM30943, AFQ95553, AFS28881, AFS28882, AFU52934, AFU72533, AFV61775, AFV61776, AFV70811, AGA93117, AGC08387, AGH25888, AGH25932, AGY36217, AHB50526, AHB52332, AHU88030, AIU97943, AIY55492, AJO62057, AKU47816, ALW54844, ALX81666, AMC38503, AMR93994, ANA48365, ANH22633, ANH79182, ANY95172, AOC37868, APX54983, AQU42721, ASB15793, ASC55665, ASK09431, ASK09432, ATY46581, AUB51246, AUD37401, AUP47462, AVQ05260, AXK90538, AYA60486, AZB50213, AZL49328, BAA00398, BAA01726, BAA12099, BAA22702, BAA25147, BAA86288, BAE78404, BAE87001, BAJ10980, BAJ19142, BAN63167, BAV17838, BBD33993, BBD74024, BBP49732, CAA04929, CAA27720, CAA47905, CAA66281, CAA81549, CAB43195, CAB51641, CAB75857, CAC17411, CAC82225, CAC83052, CAC84437, CAC85226, CAC86160, CAC87085, CAD24792, CAD24793, CAD53318, CAD92110, CAH65461, CAI23782, CAJ01678, CAJ43612, CAJ57401, CAJ57715, NP_870995, QBS16347, QDA01852, QDC21205, QDC21209, QHO60634, QIA62007, QOD42425, QRG34965, YP_022751  ^d^ One accession has an alignment gap at this position and was discarded from statistics | | | | | | | | | |
