# Supplementary material for: AlkB RNA demethylase homologues and N 6 ‐methyladenosine are involved in Potyvirus infection
Source: Mol Plant Pathol. 2022 Jun 14;23(10):1555–64. doi: 10.1111/mpp.13239 (PMC9452765; doi:10.1111/mpp.13239)
Supplement: Supplementary file 10 — Table S4 Viral AlkB phylogenetic tree in Newick format including bootstrap values, species names, (poly)protein accession numbers, and residue positions [file MPP-23-1555-s008.docx]

### Table S4. Viral AlkB phylogenetic tree in Newick format including bootstrap values, species names, (poly)protein accession numbers and residue positions

| (Escherichia_coli_BAA15995.1_64-210:3.451707354,((((((((Actinidia_seed_borne_latent_virus_AUZ97243.1_855-980:0.6569264121,Caucasus_prunus_virus_AKN08994.1_860-987:0.418686839):0.1935233706,(Cherry_mottle_leaf_virus_AAF86666.1_705-833:0.1594467418,Peach_mosaic_virus_ABA18636.1_706-834:0.2007046362)100:1.116941487)94:0.118511777,((Apricot_vein_clearing_associated_virus_CDF66416.2_514-635:0.3832151141,Karelinia_prunevirus_A_QED42807.1_878-1000:0.4402524575)87:0.1866878064,((Asparagus_virus_3_BAG12158.1_544-665:0.5852624884,Lettuce_virus_X_CAN88808.1_604-725:0.465040817)99:0.0806922532,Malva_mosaic_virus_ABG48660.1_527-649:0.6012247914)100:0.2778459752)82:0.2336774612)66:0.0060855833,((Asian_prunus_virus_1_ACU30132.1_769-900:0.1495419164,Asian_prunus_virus_2_ALT57194.1_765-896:0.1199337154)100:0.583444647,Blackberry_virus_Y_AAX87001.1_146-274:1.329228163)65:0.2482771987)35:0.2544873051,((Black_raspberry_necrosis_virus_ABC71319.1_2025-2152:0.7913975824,(Little_cherry_virus_2_AAP87783.1_915-1040:0.7673770418,((Citrus_yellow_mottle_virus_QGT76798.1_536-661:0.0726505778,Indian_citrus_ringspot_virus_AAK97522.1_537-662:0.0938182948)99:0.055356668,Citrus_yellow_vein_clearing_virus_AJO26399.1_537-662:0.0043913583)100:1.047932751)44:0.128057129)46:0.1160875657,Carrot_Ch_virus_1_AHA85534.1_709-840:0.8798594505)65:0.1167255391)77:0.0216551422,((Cherry_necrotic_rusty_mottle_virus_AAF78210.1_748-881:0.1699925473,Cherry_twisted_leaf_associated_virus_AID51400.1_746-879:0.2140194112)97:0.1502783967,Cherry_rusty_mottle_associated_virus_AGI62177.1_737-870:0.2451774707)100:0.5624125869)55:0.2793209991,(((((((Alfalfa_virus_S_ARQ03275.1_591-721:0.4231948259,Plum_bark_necrosis_stem_pitting-associated_virus_ABW81234.1_302-431:0.7291169027)85:0.1479598753,Arracacha_virus_V_ARD06099.1_617-746:0.5902530839)45:0.0961308189,Grapevine_leafroll-associated_virus_4_ACS44657.1_1675-1803:0.6336397352)32:0.0587538118,(((Alternanthera_mosaic_virus_AAX62019.1_550-681:0.2389830429,((Babaco_mosaic_virus_AUG45968.1_548-679:0.2587023613,Papaya_mosaic_virus_BAA03050.1_556-687:0.3196080674)98:0.1449735915,Senna_mosaic_virus_ANW11492.1_548-679:0.1841269879)92:0.1127997047)97:0.209318102,((French_endive_necrotic_mosaic_virus_ARF07717.1_71-199:0.1899784049,Endive_necrotic_mosaic_virus_UOF93311.1_86-214:0.2930068027)100:0.4426311977,Grapevine_leafroll-associated_virus_13_BAU68560.1_1508-1636:0.9224001295)45:0.0652047181)60:0.1405359937,Papaya_virus_A_QIM41186.1_619-747:0.4452727038)30:0.1106278594)12:0.0562279293,((Shallot_virus_X_AAA47787.1_612-740:0.4586035607,Clover_yellow_mosaic_virus_QLJ11034.1_687-816:0.6268601862)51:0.0414032081,Peach_chlorotic_mottle_virus_ABV58371.1_716-845:0.6782661083)27:0.0453760365)14:0.0408706405,(((Chrysanthemum_virus_B_CAM35753.2_749-881:0.1909874558,Chrysanthemum_virus_R_AVN98095.1_743-875:0.1680018648)100:0.4501627782,(((Currant_virus_A_ALT08067.1_627-756:0.719569153,(Garlic_yellow_virus_QED44835.1_637-766:0.5996949497,Sweet_potato_chlorotic_fleck_virus_AAS18575.1_752-882:0.7575669775)93:0.1653876246)47:0.0533420459,Helenium_virus_S_QQX32728.1_702-831:0.4208807695)55:0.1491811754,Rose_virus_A_QEV82104.1_723-855:0.7483269747)97:0.1190186975)45:0.059613646,((Grapevine_rupestris_stem_pitting-associated_virus_1_AAC35498.1_740-874:0.6180509424,Grapevine_virus_T_AXH64978.1_717-852:0.350004179)67:0.1456759804,Actinidia_virus_1_ASR91587.1_548-680:2.286468354)69:0.2995278304)59:0.1890811354)23:0.1194812249,((Blackberry_vein_banding-associated_virus_AGS48177.1_1700-1830:0.5357538251,Grapevine_leafroll-associated_virus_3_AAC40717.3_1561-1691:0.4087490561)95:0.1367904383,Blueberry_green_mosaic_associated_virus_QEH60473.1_629-757:0.5879731186)54:0.0213681883)19:0.1702679143)81:0.0772550656,(((Actinidia_virus_A_AET36885.1_613-739:0.2138370923,Actinidia_virus_B_AET36890.1_605-731:0.3198531514)96:0.0653315163,Grapevine_virus_B_CAA53196.1_607-733:0.4154423371)99:0.2091237085,(Grapevine_virus_H_ASN77903.1_606-735:0.6388558574,((((Grapevine_leafroll-associated_virus_1_AEW24401.1_1515-1645:0.3653724896,Grapevine_virus_A_CAA53182.1_605-735:0.2409175432)96:0.1138800869,(Lolium_latent_virus_ACA53374.1_701-831:0.5975327573,(((((Cactus_virus_X_AAK69579.1_571-702:0.2276468488,Zygocactus_virus_X_AAR11542.1_572-703:0.1864606556)99:0.0848499776,Papaya_virus_X_QIL68840.1_587-718:0.5025016871)96:0.103340483,Opuntia_virus_X_AAR11547.1_584-715:0.5758524097)87:0.0606478652,Schlumbergera_virus_X_AAR11537.2_574-705:0.1367658222)89:0.1049173997,Pitaya_virus_X_AFI57890.1_586-717:0.0992046186)100:0.6110764431)93:0.1551012227)56:0.0942207945,Grapevine_virus_F_AFP95341.1_615-745:0.5451822906)41:0.0493035952,(Grapevine_virus_D_AVD73311.1_608-737:0.4544120714,Grapevine_virus_J_AVI69646.1_612-741:0.2977207678)98:0.2595046062)60:0.1113379074)31:2.1654e-06)59:0.0521418915)37:0.309625829)RT; |
| --- |
