# Supplementary material for: AlkB RNA demethylase homologues and N 6 ‐methyladenosine are involved in Potyvirus infection
Source: Mol Plant Pathol. 2022 Jun 14;23(10):1555–64. doi: 10.1111/mpp.13239 (PMC9452765; doi:10.1111/mpp.13239)
Supplement: Supplementary file 11 — Table S5 m6A RNA sequencing samples and statistical results [file MPP-23-1555-s004.docx]

### Table S5. m^6^A RNA sequencing samples and statistical results

| Sample_ID | Raw_Reads | Raw_Bases | Valid_Reads | Valid_Bases |
| --- | --- | --- | --- | --- |
| Healthy1_IP | 44014942 | 6.60G | 41190256 | 6.10G |
| Healthy2_IP | 42599178 | 6.39G | 39931926 | 5.93G |
| Healthy3_IP | 39951536 | 5.99G | 37922676 | 5.55G |
| PPV1_IP | 45839764 | 6.88G | 42367946 | 6.27G |
| PPV2_IP | 42198500 | 6.33G | 38812864 | 5.74G |
| PPV3_IP | 34641152 | 5.20G | 31937662 | 4.72G |
| PVY1_IP | 50666540 | 7.60G | 47908438 | 7.05G |
| PVY2_IP | 44719236 | 6.71G | 42175216 | 6.21G |
| PVY3_IP | 46425928 | 6.96G | 43835566 | 6.46G |
| Healthy1_input | 43734580 | 6.56G | 43371906 | 6.50G |
| Healthy2_input | 56930282 | 8.54G | 56186006 | 8.37G |
| Healthy3_input | 59486524 | 8.92G | 58603838 | 8.76G |
| PPV1_input | 45995092 | 6.90G | 45284280 | 6.77G |
| PPV2_input | 44663150 | 6.70G | 44040624 | 6.59G |
| PPV3_input | 46138558 | 6.92G | 45539390 | 6.81G |
| PVY1_input | 61705944 | 9.26G | 61099756 | 9.15G |
| PVY2_input | 57360440 | 8.60G | 56791208 | 8.50G |
| PVY3_input | 64579970 | 9.69G | 63966710 | 9.58G |
