# Supplementary material for: AlkB RNA demethylase homologues and N 6 ‐methyladenosine are involved in Potyvirus infection
Source: Mol Plant Pathol. 2022 Jun 14;23(10):1555–64. doi: 10.1111/mpp.13239 (PMC9452765; doi:10.1111/mpp.13239)
Supplement: Supplementary file 12 — Table S6 m6A peak enrichment in PPV and PVY genomes [file MPP-23-1555-s001.docx]

### Table S6. m^6^A peak enrichment in PPV and PVY genomes

| Virus ^a^ | Peak | Peak_start | Peak_end | -LOG10(qvalue) |
| --- | --- | --- | --- | --- |
| PPV | Peak_1 | 3751 | 4109 | 160.16 |
| PPV | Peak_2 | 4793 | 10448 | 612.54 |
| PVY | Peak_1 | 2449 | 3819 | 240.14 |
| PVY | Peak_2 | 4061 | 4464 | 49.15 |
| PVY | Peak_3 | 5052 | 5855 | 565.82 |
| PVY | Peak_4 | 8728 | 9660 | 1741.08 |
| ^a^ The pLX-PPV viral cDNA cassette (GenBank: KY825150) was used for PPV read mapping; potato virus Y strain NTN genome (GenBank: EF026075) was used for PVY read mapping. | | | | |
