# Supplementary material for: AlkB RNA demethylase homologues and N 6 ‐methyladenosine are involved in Potyvirus infection
Source: Mol Plant Pathol. 2022 Jun 14;23(10):1555–64. doi: 10.1111/mpp.13239 (PMC9452765; doi:10.1111/mpp.13239)
Supplement: Supplementary file 13 — Table S7 Arabidopsis thaliana transcriptomic datasets used for AlkB homologue expression analysis [file MPP-23-1555-s014.docx]

### Table S7. *Arabidopsis thaliana* transcriptomic datasets used for AlkB homologue expression analysis

| Type | Source | Source_ID | Description | Reference |
| --- | --- | --- | --- | --- |
| Microarray | Genevestigator | AT-00110 | AtGenExpress: response of wild-type seedlings to seven phytohormones (IAA, zeatin, GA3, ABA, MJ, ACC, BL) | (Nemhauser et al., 2006) |
| Microarray | Genevestigator | AT-00320 | Expression data from Arabidopsis seedlings treated with salicylic acid | (Hruz et al., 2008) |
| Microarray | Genevestigator | AT-00318 | Effect of geminivirus Cabbage leaf curl virus on Arabidopsis Col-0 at 12 days post-inoculation during short-day conditions | (Ascencio-Ibáñez et al., 2008) |
| Microarray | Genevestigator | AT-00324 | Transcription profiling by of Arabidopsis leaves after infection with Potyvirus turnip mosaic virus | (Yang et al., 2007) |
| RNAseq | NCBI BioProject | PRJNA336058 | Comparative transcriptome analysis of TCV-infected wildtype and dcl1-9 mutant Arabidopsis thaliana | (Wu et al., 2016) |

References

Ascencio-Ibáñez, J.T., Sozzani, R., Lee, T.-J., Chu, T.-M., Wolfinger, R.D., Cella, R., et al. (2008) Global analysis of Arabidopsis gene expression uncovers a complex array of changes impacting pathogen response and cell cycle during geminivirus infection. *Plant Physiology*, 148, 436–454. https://doi.org/10.1104/pp.108.121038.

Hruz, T., Laule, O., Szabo, G., Wessendorp, F., Bleuler, S., Oertle, L., et al. (2008) Genevestigator v3: a reference expression database for the meta-analysis of transcriptomes. *Advances in Bioinformatics*, 2008, 420747. https://doi.org/10.1155/2008/420747.

Nemhauser, J.L., Hong, F. & Chory, J. (2006) Different plant hormones regulate similar processes through largely nonoverlapping transcriptional responses. *Cell*, 126, 467–475. <https://doi.org/10.1016/j.cell.2006.05.050>.

Wu, C., Li, X., Guo, S. & Wong, S.-M. (2016) Analyses of RNA-Seq and sRNA-Seq data reveal a complex network of anti-viral defense in TCV-infected *Arabidopsis thaliana*. *Scientific Reports*, 6, 36007. https://doi.org/10.1038/srep36007.

Yang, C., Guo, R., Jie, F., Nettleton, D., Peng, J., Carr, T., et al. (2007) Spatial analysis of *Arabidopsis thaliana* gene expression in response to *Turnip mosaic virus* infection. *Molecular plant-microbe interactions*, 20, 358–370. https://doi.org/10.1094/mpmi-20-4-0358.
