# Supplementary material for: AlkB RNA demethylase homologues and N 6 ‐methyladenosine are involved in Potyvirus infection
Source: Mol Plant Pathol. 2022 Jun 14;23(10):1555–64. doi: 10.1111/mpp.13239 (PMC9452765; doi:10.1111/mpp.13239)
Supplement: Supplementary file 14 — Table S8 Fold changes of AlkB homologue expression in the Arabidopsis thaliana transcriptomic datasets analysed [file MPP-23-1555-s007.docx]

### Table S8. Fold changes of AlkB homologue expression in the *Arabidopsis thaliana* transcriptomic datasets analysed

| AGI | ID | Log2(FC) ^a^ | | | | | | | | | | |
| --- | --- | --- | --- | --- | --- | --- | --- | --- | --- | --- | --- | --- |
|  |  | TuMV | TCV | CaLCuV | SA | JA | ABA | ACC | GA | IAA | CK | BL |
| AT1G11780 | ALKBH1A | 0.30 | -0.07 | 0.32 | -0.10 | 0.20 | -0.20 | 0.37 | 0.10 | 0.14 | 0.41 | 0.24 |
| AT3G14140 | ALKBH1B | n.a. | 0.13 | n.a. | n.a. | n.a. | n.a. | n.a. | n.a. | n.a. | n.a. | n.a. |
| AT3G14160 | ALKBH1C | 0.02 | -0.03 | 0.24 | 0.65 | 0.09 | -0.25 | 0.24 | -0.01 | 0.21 | 0.18 | 0.07 |
| AT5G01780 | ALKBH1D | -0.26 | 0.23 | -0.35 | -0.49 | 0.18 | 0.23 | 0.23 | 0.05 | -0.03 | 0.10 | 0.11 |
| AT2G22260 | ALKBH2 | -0.15 | -0.08 | -0.42 | -0.16 | 0.10 | -0.19 | 0.13 | -0.02 | 0.14 | -0.13 | 0.08 |
| AT4G20350 | ALKBH6 | -0.14 | 0.09 | -0.06 | 0.08 | -0.02 | -0.12 | 0.01 | 0.04 | 0.16 | 0.07 | 0.15 |
| AT1G31600 | ALKBH8A | 0.20 | 0.19 | 0.32 | -0.21 | 0.13 | -0.09 | 0.06 | -0.09 | 0.12 | -0.22 | 0.05 |
| AT4G02485 | ALKBH8B | -0.25 | 0.31 | 0.18 | -0.31 | 0.07 | -0.13 | -0.01 | 0.10 | 0.05 | 0.06 | -0.17 |
| AT1G48980 | ALKBH9A | -0.21 | n.a. | -0.22 | -0.01 | -0.02 | -0.16 | -0.05 | -0.02 | -0.17 | -0.09 | -0.19 |
| AT2G17970 | ALKBH9B | -0.16 | 0.17 | 0.26 | -0.11 | -0.07 | -0.40 | -0.08 | -0.15 | 0.08 | -0.06 | -0.20 |
| AT4G36090 | ALKBH9C | n.a. | 0.32 | n.a. | n.a. | n.a. | n.a. | n.a. | n.a. | n.a. | n.a. | n.a. |
| AT2G48080 | ALKBH10A | -0.19 | 0.43 | -0.32 | -0.16 | -0.06 | -0.61 | -0.33 | -0.04 | -0.57 | -0.05 | 0.29 |
| AT4G02940 | ALKBH10B | -0.05 | 0.10 | 0.90 | 1.09 | 0.80 | 0.35 | -0.26 | -0.07 | -0.22 | -0.15 | -0.30 |
| AT1G14710 | ALKBH10C | 0.22 | -0.64 | 0.14 | -0.39 | -0.27 | -0.21 | -0.05 | 0.09 | -0.13 | 0.47 | 0.04 |
| AT2G14610 | PR-1 | 3.59 | 1.35 | 6.99 | 8.40 | 0.23 | 0.24 | 1.34 | 1.29 | 1.11 | 0.87 | 0.77 |
| ^a^ TuMV, turnip mosaic virus; TCV, turnip crinkle virus; CaLCuV, cabbage leaf curl virus; SA, salicylic acid; JA, methyl jasmonate; ABA, abscisic acid; ACC, aminocyclopropane-1-carboxylic acid; GA, gibberellic acid; IAA, indole-3-acetic acid; CK, zeatin; BL, brassinolide; n.a., not detected. | | | | | | | | | | | | |
