# Supplementary material for: AlkB RNA demethylase homologues and N 6 ‐methyladenosine are involved in Potyvirus infection
Source: Mol Plant Pathol. 2022 Jun 14;23(10):1555–64. doi: 10.1111/mpp.13239 (PMC9452765; doi:10.1111/mpp.13239)
Supplement: Supplementary file 15 — Table S9 Phylogenetic tree of plant AlkB homologues in Newick format including bootstrap values, protein accession numbers, IDs, and residue positions [file MPP-23-1555-s015.docx]

### Table S9. Phylogenetic tree of plant AlkB homologues in Newick format including bootstrap values, protein accession numbers, IDs, and residue positions

| (AT1G11780.1_ALKBH1A_1-345:1.4637397947,((AT3G14140.1_ALKBH1B_1-473:0.3681193277,AT3G14160.1_ALKBH1C_1-455:0.2615442353)93:0.4304863867,AT5G01780.2_ALKBH1D_1-442:0.8326543346)100:1.2108136022,((AT2G22260.1_ALKBH2_1-314:1.9195175850,(AT4G20350.2_ALKBH6_1-241:1.7892137475,(AT1G31600.1_ALKBH8A_1-431:1.0806886523,AT4G02485.1_ALKBH8B_1-226:1.2603980633)37:0.2190012345)76:0.6444334000)59:0.2788428481,(((((AT1G48980.1_ALKBH9A_1-331:0.3881443195,AT2G17970.1_ALKBH9B_1-507:0.1642855265)62:0.1473967814,AT4G36090.3_ALKBH9C_1-520:0.2273262551)55:0.1173499277,QPC97718.1_NbALKB2_1-540:0.4575719929)64:0.1963340530,QYU76043.1_NbALKB1_1-439:0.6165964538)100:0.8702162152,((AT2G48080.1_ALKBH10A_1-438:0.3927226554,AT4G02940.1_ALKBH10B_1-569:0.2905122588)100:0.8006959872,AT1G14710.1_ALKBH10C_1-601:0.7636077845)98:0.5390890144)99:0.7067279351)88:0.7414675293); |
| --- |
