# Supplementary material for: AlkB RNA demethylase homologues and N 6 ‐methyladenosine are involved in Potyvirus infection
Source: Mol Plant Pathol. 2022 Jun 14;23(10):1555–64. doi: 10.1111/mpp.13239 (PMC9452765; doi:10.1111/mpp.13239)
Supplement: Supplementary file 16 — Table S10 PCR primers used in the study [file MPP-23-1555-s012.docx]

### Table S10. PCR primers used in the study

| ID | Sequence (5'-3') | Use |
| --- | --- | --- |
| NbALKB1_F | ATGGCCGGAGATTTTTGCCGC | Gene cloning |
| NbALKB1_R | TTATCTCTGACTTCTACGGTTGG |  |
| NbALKB2_F | ATGTCCGAGTCCGACCAGCAGCG |  |
| NbALKB2_R | TCAATTTTCTGTATTCACGTTAAACC |  |
| NbALKB1-q_F | GGTCGGTGCTCATTCTCAAT | Transcript quantification |
| NbALKB1-q_R | GCTCAGGGTCAGGAGTATTTG |  |
| NbALKB2-q_F | CCAGCAGCGAACTCAGAAG |  |
| NbALKB2-q_R | AGAGGTCACGGGAGAGAAAG |  |
| NbUB1-q_F | TCCAGGACAAGGAGGGTATCC |  |
| NbUB1-q_R | GTCAGCCAAGGTCCTTCCATCC |  |
| NbPSMD1-q_F | GCTGCTCTAGGAACTGCTGATGA |  |
| NbPSMD1-q_R | TACCTTGTGCCAAACCCCTGAT |  |
| PPV-q_F | ATCCCCGAATATCAATGGAATGTGG |  |
| PPV-q_R | TATACGCTTCAGCCACGTTACT |  |
| NbALKB1-TRV2_F | TGCTCTAGACAGTTCGCAGGATTTCATGG | VIGS construct assembly |
| NbALKB1-TRV2_R | CCGCTCGAGGTGTATGAGCATAAGAATTCAGCC |  |
| NbALKB2-TRV2_F | TGCTCTAGATTGGAGAGGCAACTACAAGTG |  |
| NbALKB2-TRV2_R | CCGCTCGAGTTCTGTTTTTGGTTTACTCTCCGG |  |
